# Mathematical model of TGF-*β*signalling: feedback coupling is consistent with signal switching

**DOI:** 10.1186/s12918-017-0421-5

**Published:** 2017-04-13

**Authors:** Shabnam Khatibi, Hong-Jian Zhu, John Wagner, Chin Wee Tan, Jonathan H. Manton, Antony W. Burgess

**Affiliations:** 1 Electrical and Electronic Engineering Department, The University of Melbourne, Parkville, Victoria, 3010 Australia; 2 Department of Surgery (RMH), The University of Melbourne, Parkville, Victoria, 3050 Australia; 3 IBM Research Collaboratory for Life Sciences–Melbourne, Victorian Life Sciences Computation Initiative, 87 Grattan Street, Victoria, 3010 Australia; 4 IBM Research–Australia, 204 Lygon Street Level 5, Carlton, Victoria, 3053 Australia; 5 The Walter and Eliza Hall Institute of Medical Research (WEHI), 1G Royal Parade, Parkville, Victoria, 3052 Australia; 6 Department of Medical Biology, The University of Melbourne, 1G Royal Parade, Parkville, Victoria, 3052 Australia

**Keywords:** TGF-*β* signalling, Mathematical modelling, Feedback coupling, Time-delay, Reduction, Rapid equilibrium assumption, Cancer, Signal switching

## Abstract

**Background:**

Transforming growth factor *β* (TGF-*β*) signalling regulates the development of embryos and tissue homeostasis in adults. In conjunction with other oncogenic changes, long-term perturbation of TGF-*β* signalling is associated with cancer metastasis. Although TGF-*β* signalling can be complex, many of the signalling components are well defined, so it is possible to develop mathematical models of TGF-*β* signalling using reduction and scaling methods. The parameterization of our TGF-*β* signalling model is consistent with experimental data.

**Results:**

We developed our mathematical model for the TGF-*β* signalling pathway, i.e. the RF- model of TGF-*β* signalling, using the “rapid equilibrium assumption” to reduce the network of TGF-*β* signalling reactions based on the time scales of the individual reactions. By adding time-delayed positive feedback to the inherent time-delayed negative feedback for TGF-*β* signalling. We were able to simulate the sigmoidal, switch-like behaviour observed for the concentration dependence of long-term (> 3 hours) TGF-*β* stimulation. Computer simulations revealed the vital role of the coupling of the positive and negative feedback loops on the regulation of the TGF-*β* signalling system. The incorporation of time-delays for the negative feedback loop improved the accuracy, stability and robustness of the model. This model reproduces both the short-term and long-term switching responses for the intracellular signalling pathways at different TGF-*β* concentrations. We have tested the model against experimental data from MEF (mouse embryonic fibroblasts) WT, SV40-immortalized MEFs and Gp130 ^*F*/*F*^ MEFs. The predictions from the RF- model are consistent with the experimental data.

**Conclusions:**

Signalling feedback loops are required to model TGF-*β* signal transduction and its effects on normal and cancer cells. We focus on the effects of time-delayed feedback loops and their coupling to ligand stimulation in this system. The model was simplified and reduced to its key components using standard methods and the rapid equilibrium assumption. We detected differences in short-term and long-term signal switching. The results from the RF- model compare well with experimental data and predict the dynamics of TGF-*β* signalling in cancer cells with different mutations.

**Electronic supplementary material:**

The online version of this article (doi:10.1186/s12918-017-0421-5) contains supplementary material, which is available to authorized users.

## Background

TGF-*β* is a member of the transforming growth factor superfamily, which also includes other growth factors such as bone morphogenetic proteins, Mullerian inhibitory substance, activin, inhibin and Nodal [1–3]. Each family member controls a broad range of cellular processes, such as differentiation, proliferation, migration, life span and apoptosis [1, 4]. TGF-*β* is secreted in an inactive form and sequestered in the extracellular matrix, but once activated by serine and metalloproteinases [5] TGF-*β* is released and binds to the cell surface to form TGF-*β* receptor complexes. The active ligand:receptor complex then initiates the intracellular signalling that leads to SMAD activation (phosphorylation) and nucleocytoplasmic shuttling and, eventually, to gene responses in the nucleus [6, 7].

Recent studies indicate that TGF-*β* concentration, stimulation time, cell type and even the percentage of active signalling components within cells can influence the gene responses, giving a multi-functional aspect to TGF-*β* signaling [2, 8]. This is of particular interest in colon cancer, where SMAD signalling is a critical pathway controlling the transition of normal epithelial cells to cancerous cells [3, 8–11]. In spite of the myriad studies on the TGF-*β* signalling pathway, there are still many unanswered questions concerning the impact of TGF-*β* signalling at different stages of cancer cell progression [12]. In particular, there are two opposing reactions of cancer cells to TGF-*β*: the proliferation of cancer cells at an early-stage is inhibited by TGF-*β* [13], yet at more advanced stages of malignancy, proliferation of cancer cells is stimulated by this cytokine [14].

Although many of the TGF-*β* signalling components were discovered decades ago [15], the quantitation, dynamics and locations of the signalling components that occur within hours of TGF-*β* stimulation [16–22] have been more difficult to interpret. Consequently, mathematical models of TGF-*β* signalling have been developed [2, 16–21, 23, 24]. In a comprehensive model of TGF-*β* signalling, Zi et al. [22] aim to explain the high cooperativity and discontinuous cellular responses to TGF-*β* in terms of switch-like behavior arising from ligand depletion. However, these models did not include the feedback mechanisms known to regulate the TGF-*β* system, in particular feedback through SMAD7, a key inhibitor in TGF-*β* signal transduction [25]. Furthermore, SMAD7 is an important component for mediating the crosstalk between TGF-*β* signal transduction and other cytokine signalling pathways such as IL-6 or IL-11 [10].

**Fig. 1:**
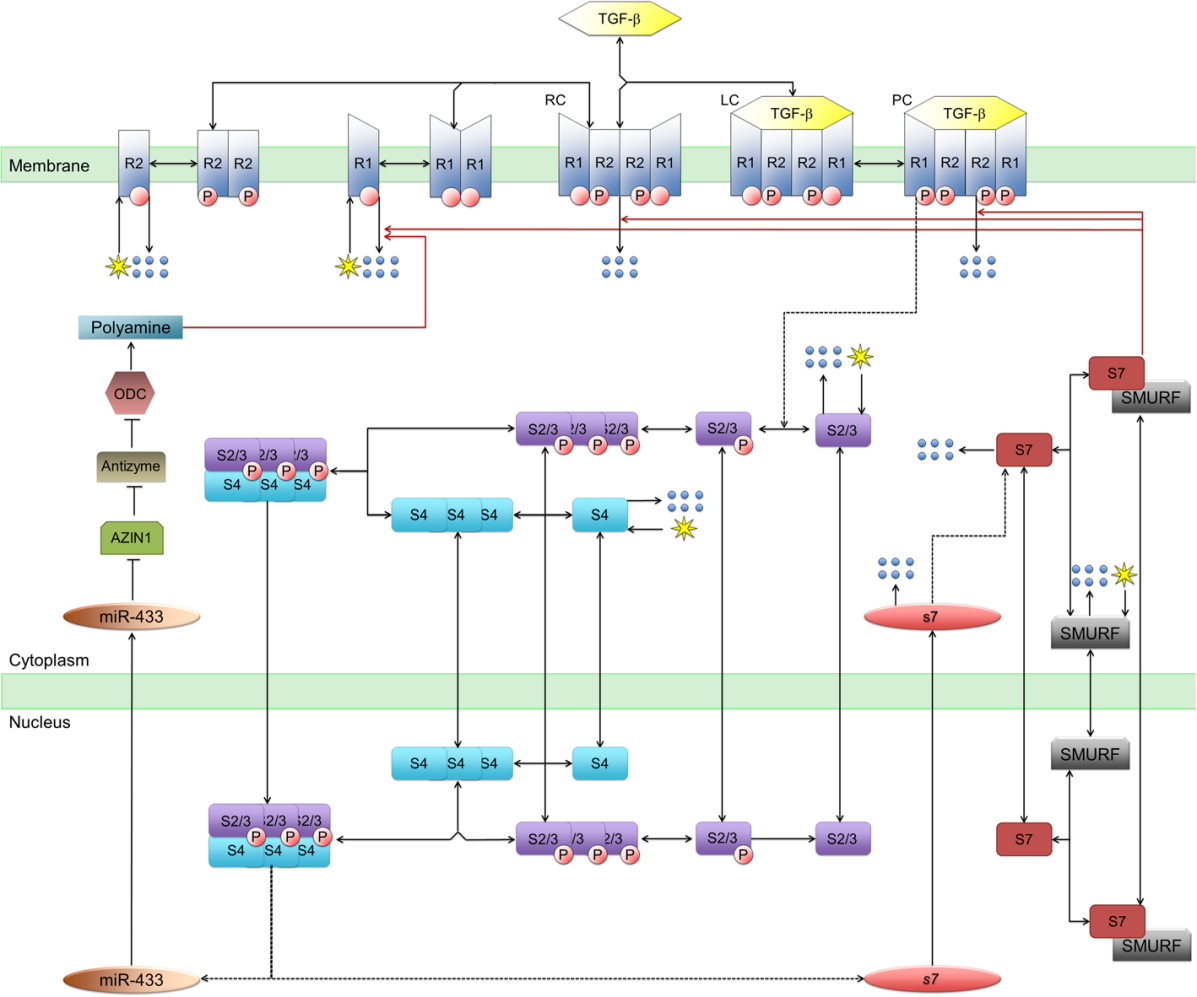
The full TGF-*β* signalling biological model. Potential phosphorylation sites of the receptors are specified with empty circles attached to R1 and R2 components. Arrows pointing to 6 blue dots represent degradation process. The stars indicate the production processes for specific proteins on the membrane and in the cytoplasm. The red solid arrows originating from SMAD7/Smurf apply negative and/or positive feedback on the receptor components of the membrane. Oval-shaped components written in small letters represent micro-RNAs. In this figure, *S* represents the SMAD proteins. Note that the arrow from ODC to polyamine shows an stimulatory reaction rather than conversion

The Zi model [22] also lacks the more recently discovered positive feedback loop in TGF-*β* signalling that acts by suppressing Azin1 via the microRNA miR-433 [26]. Azin1 promotes polyamine synthesis [26, 27], which suppresses TGF-*β* signalling [26, 28–30]. Azin1 inhibits antizyme, thus preventing the degradation of ornithine decarboxylase (ODC) [26, 27]. ODC is essential for the biosynthesis of polyamines [26, 27] (see Fig. 1). Interestingly, over-expression of Azin1 suppresses the expression of TGF-*β* and its Type 1 receptor [26]. The miR-433:Azin1:Antizyme:ODC reactions appears to induce a positive feedback on TGF-*β* signalling [26].

It is likely that these feedback loops will produce both cooperativity and switch-like behavior, even in the absence of ligand depletion [31–35]. The modelling of feedback loops requires the introduction of time-delays due to the extended time scales of the reactions. This is typically found in cellular signalling systems which involve gene regulation, protein synthesis and for the shuttling of signalling components between subcellular compartments [31–33].

As a prelude to improving our understanding of the TGF-*β* signalling system we have developed a new mathematical model which incorporates negative feedback control via SMAD signalling, positive feedback via Azin1 and appropriate time-delays for specific reactions [25, 36]. We started the modelling process by incorporating all of the reactions involved in TGF-*β* and SMAD signalling, including the feedback loops and time-delays. We then used the rapid equilibrium assumption to produce a simpler system that is more amenable to robust mathematical analysis and numerical simulation (section “Mathematical Model for TGF-*β* Signalling” in “Additional file 1”) [37]. The reduction methods were applied to the TGF-*β* signalling system in two steps, resulting in a semi-reduced mode and the RF- model. The RF- model allows us to characterise the system both at the steady-state and during the transient dynamics in response to TGF-*β* signals. It should be noted that the activation of TGF-*β* receptors also stimulates the MAPK (Mitogen-activated protein kinases) [38–41] and P38 [40–42] systems, which will influence the responses of late-stage cancer cells. The predictions from the proposed model are compared with published experimental data [22] and new experimental data from our laboratory.

## Development of the TGF-*β* signalling model

The TGF-*β* receptor complex is a tetramer comprised of Type 1 and Type 2 receptors that, upon TGF-*β* binding, becomes activated via autophosphorylation [1, 43, 44] (Fig. 1). The activated TGF-*β* receptor complex is then internalized [45, 46], where it phosphorylates and activates SMAD2/3 [44]. Activated SMAD2/3 then forms homotrimers, which bind to SMAD4 homotrimers. The heterotrimers (hexamers) are imported into the nucleus [47]. The phosphorylated SMAD2/3:SMAD4 complex functions as a transcription factor that upregulates a number of target genes, including Jun, Fos, SNAIL1 and SMAD7; the last of these target genes, SMAD7 is a known inhibitor of TGF-*β* Type 1 receptors and TGF-*β* receptor signalling [25, 47, 48]. The detailed reactions for this signalling system are summarized in Fig. 1.

Signalling systems like the TGF-*β* pathway can be modelled using ordinary differential equations which describe the concentration changes of the various cellular components (e.g TGF-*β* receptors, SMAD4) as a function of time [49]. TGF-*β* receptor activation starts with the dimerization of both components (TGF-*β* receptor type 1 and 2, called respectively R1 and R2). dimers are vital for the signalling processes [50, 51]. The R2 dimer binds to the R1 dimer, resulting in the receptor complex RC. The RC complex binds TGF-*β* dimers present in the medium around TGF-*β*:RC complex (LC) contains all the components essential for signalling, however, the R1 s are not yet activated (phosphorylated), i.e. LC is not the membrane transducer of the exogenous TGF-*β* signal. Signalling requires ligand stimulated phosphorylation of R1 by R2 to produce a phosphorylated ligand-receptor complex (PC in Fig. 1). PC an intermediate component caused by the binding of the ligand (TGF-*β*) to the R1 monomers. A degradation reaction for LC is not necessary as LC is PC and degraded through PC.

After phosphorylation of SMAD2/3, the SMADs oligomerize to form the (PSMAD2/3)_3_:(SMAD4)_3_ complex [52]. (PSMAD2/3)_3_:(SMAD4)_3_ translocates to the nucleus, stimulating the SMAD7 gene and the expression of the miR-433 microRNA [26, 53]. The SMAD7 mRNA is translated and eventually the SMAD7/SMURF complex accelerates the degradation of the R1-associated membrane components [54, 55]. Although receptor dimerization of type1 and 2 receptors on the membrane are reported to occur in different orders [56–59], the short time scale of the receptor dimerization reactions means that the dimerization order does not change the steady-state receptor output for the TGF-*β*: TGF-*β*R signalling system.

In considering the development of a model for a signalling pathway, it is important to consider all of the processes associated with the dynamics, activation, transfer, maintenance or damping of the signal. Some signalling processes are triggered rapidly and reach a new steady-state within minutes. Other processes require hours or even days to reach new steady-states. In our modelling process we defined as many processes as is practical (to produce a detailed model) and then studied the contributions of the different processes (reactions) to the regulation of specific components between 5 minutes (“short”-term) and three hours (“long”-term). Where particular reactions reach equilibrium rapidly, we introduced several “fast” reactions where only the final concentration of the “fast” reaction products appear in the “slow” equations as functions of the substances (rapid equilibrium assumption). N.B. the rapid equilibrium assumption is a special form of quasi steady-state approximation (QSSA) which is often used in the context of time scale separation (see [60] for a review). In order to compensate for the elimination of the “fast” reactions, time-delays are used in the RF- model. The time delays are explained in more detail in the next section. We tested the effectiveness of the model with a reduced number of equations (reduced model) for simulating the expected concentration of SMAD2 and Phospho-SMAD2 at both short times (<3 hour) and long times (>6 hour). SMAD3 plays a crucial role in regulating SMAD7 [61, 62] and miR-433 [26] and stimulating the negative and positive feedback loops. However, due to similar dynamics for SMAD2 and SMAD3 inside the cell, it is reasonable to use measurements of Phospho-SMAD2 as the output of the TGF-*β* signalling system.

### Semi-reduced model of TGF-*β* signalling

**Fig. 2:**
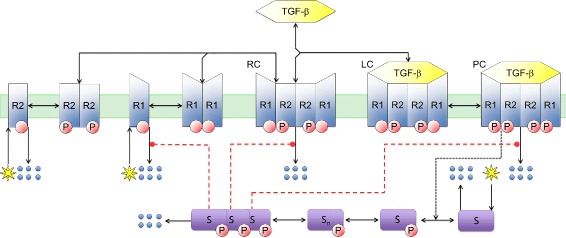
The semi-reduced TGF-*β* signal transduction reactions. The red dashed lines which originate from phosphorylated SMAD trimer indirectly regulate the receptor levels. All the reactions from trimerization of phospho-SMAD2/3 to SMAD7 transcription and translation are reduced to the red dashed lines (see Fig. 1 for clarification). The dotted ends of red dashed lines show that included reactions could lead to both inhibition and stimulation of their targeting reactions (demonstrating negative and positive feedback effects). In this figure S is specifically used for SMAD2/3

In order to reduce the number of intracellular reactions involving in TGF-*β* signaling, we have focused on the receptor components and then the direct interactions of the critical receptor components with the SMADs at the membrane. We considered the reduction process of the TGF-*β* signalling system in two steps: first by developing a semi-reduced model and second reducing it further to the RF- model. The semi-reduced model of TGF-*β* signalling is shown in Fig. 2.

We reduced the SMAD signalling interactions (e.g. nucleocytoplasmic shuttling of activated SMAD complexes and transcription and translation of feedback-associated proteins, such as SMAD7 and miR-433) to a single ligand dependent feedback loop that is regulated by the levels of the PSMAD trimer, (S)_3_. For SMAD activation of transcription, an intermediate step, S_n_, was added to mimic the nuclear accumulation of phosphorylated SMAD. These steps simplify the initial modelling equations and include negative and positive feedback loops. The two feedback loops for TGF-*β* signalling are both the result of sequences of back-to-back, coupled reactions (see Fig. 1). Each of the intracellular processes happens at specific locations, within a specific time interval and at defined kinetic rates. In order to simulate all the cytoplasmic and nuclear reactions associated with the feedback loops significant time-delays need to be incorporated into the model for TGF-*β* signalling.

In programming from the full set of reactions (Fig. 1) to the semi-reduced model (Fig. 2) several assumptions were necessary. Primarily, the component S is used to represent the initial states of the SMAD proteins. Since both SMAD2 and 3 follow similar dynamics, we assigned the single component S to represent both proteins. ${\hat {\text {S}}}$ replaces all the phoshorylated SMAD2/3 in the cytoplasm, while the nuclear PSMAD3 is represented by S_n_. SMAD4 is the common-mediator SMAD that participates in the TGF-*β* signalling by interacting with PSMAD2/3. Therefore, it is possible to incorporate the role of SMAD4 in ${\hat {\text {S}}}$. The total (PSMAD2/3) _3_.(SMAD4) _3_ concentration is represented by (S)_3_ in Fig. 2. The negative feedback cascade via SMAD7 (S _7_) is initiated from the transcriptional SMAD complex (S_n_) and is represented by the (S)_3_ component. However, (S)_3_ is represented as a dimer in the negative feedback equations in order to simulate the SMAD7:SMURF interaction.

**Fig. 3:**
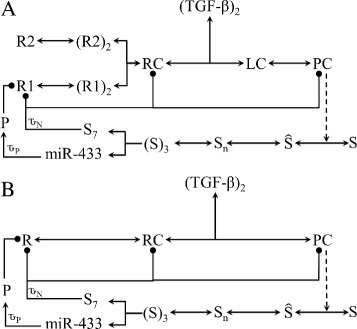
TGF-*β* receptor signalling system. **a** The schematic semi-reduced model, TGF-*β* signal transduction. TGF and ${\hat {\text {S}}}$ + 3 (S)_3_ represent the input and the output of the model. **b** A Simplified Model of TGF-*β* signal transduction. TGF-*β* and $\hat {\text {S}}+ \text {S}_{\text {n}} + 3 \text {(S)}_{\text {3}}$ represent the input and the output of the model. Both positive and negative feedback loops are indicated by dot-ended solid lines emerging from (S)_3_. *τ*_*P*_ and *τ*_*N*_ represent time-delays incorporated in the positive and negative feedback loops, respectively

The positive feedback loop is caused by a chain of biochemical reactions which are triggered by nuclear (PSMAD2/3) _3_.(SMAD4) _3_ [52]. These Azin1:Antizyme:ODC:Polyamine associated reactions are represented via a single intermediate inhibitor P. In Fig. 3 both the positive and negative feedback loops are indicated with a dot-terminated solid line emerging from miR-433 and S _7_.

According to the semi-reduced model shown in Fig. 2, the receptor associated reactions can be represented by: 
1


where ∗ represents the production process of specific proteins and ::: represents the proteosomal degradation processes. Corresponding delay differential equations describing all of the reactions associated with semi-reduced TGF-*β* signal transduction are (Fig. 2):

2 $$ {{\begin{aligned} \frac{\text{d[R1]}}{\text{d}t} &= {v_{1}} - {k_{1}} [\text{R1}] - {k_{1}^{\text{f-}}} [\text{N}]^{2} \frac{[\text{R1}]}{[\text{R1}]+{K}}- \\ &\quad 2{k_{1}^{+}} [\text{R1}] [\text{R1}] + 2{k_{1}^{-}}[(\text{R1})_{2}] - {k_{1}^{\text{f+}}} [\text{P}] \frac{[\text{R1}]}{[\text{R1}]+{K}}\\ \frac{\text{d}[\text{R2}]}{\text{dt}} &= {v_{2}} - {k_{2}} [\text{R2}] - 2{k_{2}^{+}} [\text{R2}] [\text{R2}] + 2 {k_{2}^{-}}[(\text{R2})_{2}]\\ \frac{\text{d}[(\text{R1})_{2}]}{\text{dt}} &= {k_{1}^{+}} [\text{R1}] [\text{R1}] - {k_{1}^{-}} [(\text{R1})_{2}] - {k_{\text{RC}}^{+}} [(\text{R1})_{2}] [(\text{R2})_{2}]\\ &\quad+ {k_{\text{RC}}^{-}} [\text{RC}] \\ \frac{\text{d}[(\text{R2})_{2}]}{\text{dt}} &= {k_{2}^{+}} [\text{R2}] [\text{R2}] - {k_{2}^{-}} [(\text{R2})_{2}] - {k_{\text{RC}}^{+}} [(\text{R1})_{2}] [(\text{R2})_{2}]\\ &\quad+ {k_{\text{RC}}^{-}} [\text{RC}] \\ \frac{\text{d}[\text{RC}]}{\text{dt}} &= {k_{\text{RC}}^{+}} [(\text{R1})_{2}] [(\text{R2})_{2}] - {k_{\text{RC}}^{-}} [\text{RC}] \\ &\quad -{k_{\text{LC}}^{+}} [\text{RC}] [(\text{TGF}-\beta)_{2}] + {k_{\text{LC}}^{-}} [\text{LC}] - {k_{\text{RC}}}[\text{RC}]\\ &\quad- {k_{\text{RC}}^{\text{f-}}} [\text{N}]^{2} \frac{[\text{RC}]}{[\text{RC}]+{K}} \\ \frac{\text{d}[\text{LC}]}{\text{dt}} &= {k_{\text{LC}}^{+}} [\text{RC}] [(\text{TGF}-\beta)_{2}] - {k_{\text{LC}}^{-}} [\text{LC}] - {k_{\text{PC}}^{+}} [\text{LC}]\\ &\quad+ {k_{\text{PC}}^{-}} [\text{PC}] \\ \frac{\text{d}[\text{PC}]}{\text{dt}} &= {k_{\text{PC}}^{+}} [\text{LC}] - {k_{\text{PC}}^{-}} [\text{PC}] - {k_{\text{PC}}} [\text{PC}]\\ &\quad - {k_{\text{PC}}^{\text{f-}}} [\text{N}]^{2} \frac{[\text{PC}]}{[\text{PC}]+{K}} \frac{\text{d}[\text{S}]}{\text{dt}} &= {v_{\text{S}}} - {k_{\text{S}}} [\text{S}] - {k_{\text{S}}^{+}} [\text{PC}] \frac{[\text{S}]}{[\text{S}]+{K_{\text{S}}}}+{k_{\text{S}}^{-}} [{\hat{\text{S}}}] \\ \frac{\text{d}[{\hat{\text{S}}}]}{\text{dt}} &= {k_{\text{S}}^{+}} [\text{PC}] \frac{[\text{S}]}{[\text{S}]+{K_{\text{S}}}}-{k_{\text{S}}^{-}} [{\hat{\text{S}}}] - {k_{\hat{\text{S}}}}[{\hat{\text{S}}}]\\ &\quad - {k_{n}^{+}} [{\hat{\text{S}}}] + {k_{n}^{-}} [\text{S}_{\text{n}}] \\ \frac{\text{d}[\text{S}_{\text{n}}]}{\text{dt}} &= {k_{n}^{+}} [{\hat{\text{S}}}] - {k_{n}^{-}} [\text{S}_{\text{n}}] - 3 {k_{3}^{+}} [\text{S}_{\text{n}}]^{3} + 3 {k_{3}^{-}} [(\text{S})_{3}] \\ \frac{\text{d}[(\text{S})_{3}]}{\text{dt}} &= {k_{3}^{+}} [\text{S}_{\text{n}}]^{3} - {k_{3}^{-}} [(\text{S})_{3}] - {k_{3}} [(\text{S})_{3}] \end{aligned}}}  $$

where [P]=*K*_I_^2^/(*K*_I_^2^+[(S)_3_(*t*−*τ*_*P*_)]^2^) and [N]=[(S)_3_](*t*−*τ*_*N*_), the positive and negative feedback intermediate components, respectively (see Fig. 1 for definitions of the components).

## RF - model of TGF-*β* signalling

The reduced model approximates TGF-*β* signalling with 6 differential equations. It is assumed that the R1, and R2 dynamics are similar, hence the individual components were replaced by a receptor block, R. R then become dimerized to form RC. LC and PC are combined in one parameter, i.e. PC, since they approximately follow the same kinetics. The reactions describing the receptor interactions and the initial SMAD changes are: 
3


Although some cooperativity within the system originates from the several dimer and trimer reactions on the membrane, in the cytosol and in the nucleus, the most critical cooperativity associated with the TGF-*β* induced signalling reactions comes from the trimerization of the Phosphorylated SMAD3, the binding of these oligomers to the SMAD4 trimer and the consequential stimulation of miR-433 and SMAD7 transcription. It should be noted that the trimerization of Phospho-SMADs influences both the positive and negative feedback loops (see Fig. 1).

Figure 3 describes the reaction framework we used to produce the RF- model for simulating TGF-*β* signalling and how it is derived from the semi-reduced model. The key components in the RF- model are specified in Fig. 3. This reduction/simplification method retains all of the critical components of the signalling pathways.

The set of delayed differential equations which describe the RF- model is introduced in Eq. 4. We have named this model RF- model of TGF-*β* signalling since “R” indicates that the model is “reduced” and “F” emphasizes that the positive and negative “feedback” loops are considered in the RF- model. Initially, the time-delays and amplitudes of the positive and negative feedback loops (*τ*_*P*_ and *τ*_*N*_) are assumed to be identical, however as shown in the supplementary results, it is feasible to adjust these parameters when appropriate experimental data is available.

4 $$ \begin{aligned} \frac{\mathrm{d}[\mathrm{R}]}{\mathrm{d}t} &= {v_{1}} -{k_{1}} [\mathrm{R}] - 2{k_{\text{RC}}^{+}}[\mathrm{R}]^{2} + 2{k_{\text{RC}}^{-}}[\text{RC}]\\ &\quad - {k_{1}^{\text{f+}}}[\mathrm{P}] \frac{[\mathrm{R}]}{[\mathrm{R}]+{K}} - {k_{1}^{\text{f}-}}[\mathrm{N}]^{2} \frac{[\mathrm{R}]}{[\mathrm{R}] + {K}} \\ \frac{\mathrm{d}[\text{RC}]}{\mathrm{d}t} &= {k_{\text{RC}}^{+}}[\mathrm{R}]^{2} - {k_{\text{RC}}^{-}}[\text{RC}] - {k_{\text{RC}}} [\text{RC}]\\ &\quad - {k_{\text{PC}}^{+}}[(\text{TGF}-\beta)_{2}][\text{RC}] \\ &\quad + {k_{\text{PC}}^{-}} [\text{PC}] - {k_{\text{RC}}^{\text{f}-}}[\mathrm{N}]^{2} \frac{[\text{RC}]}{[\text{RC}] + {K}} \\ \frac{\mathrm{d}[\text{PC}]}{\mathrm{d}t} &= {k_{\text{PC}}^{+}} [(\text{TGF}-\beta)_{2}][\text{RC}] - {k_{\text{PC}}^{-}} [\text{PC}]\\ &\quad - {k_{\text{PC}}} [\text{PC}] - {k_{\text{PC}}^{\text{f}-}}[\mathrm{N}]^{2} \frac{[\text{PC}]}{[\text{PC}] + {K}} \\ \frac{\mathrm{d}[\mathrm{S}]}{\mathrm{d}t} &= {v_{\mathrm{S}}} - {k_{\mathrm{S}}}[\mathrm{S}] - {k_{\mathrm{S}}^{+}} [\text{PC}]\frac{[\mathrm{S}]}{[\mathrm{S}] + {K_{\mathrm{S}}}} + {k_{\mathrm{S}}^{-}}[{\hat{\mathrm{S}}}]\\ \frac{\mathrm{d}[{\hat{\mathrm{S}}}]}{\mathrm{d}t} &= {k_{\mathrm{S}}^{+}} [\text{PC}]\frac{[\mathrm{S}]}{[\mathrm{S}] + {K_{\mathrm{S}}}} - {k_{\mathrm{S}}^{-}} [{\hat{\mathrm{S}}}] - {k_{n}^{+}}[{\hat{\mathrm{S}}}]\\ &\quad + {k_{n}^{-}}[\mathrm{S}_{\mathrm{n}}] - k_{\hat{\mathrm{S}}}[{\hat{\mathrm{S}}}]\\ \frac{\mathrm{d}[\mathrm{S}_{\mathrm{n}}]}{\mathrm{d}t} &= {k_{n}^{+}}[{\hat{\mathrm{S}}}] - {k_{n}^{-}}[\mathrm{S}_{\mathrm{n}}] - {k_{\mathrm{S}_{\mathrm{n}}}} [\mathrm{S}_{\mathrm{n}}] \end{aligned}  $$

where again, [(S)_3_]=[S_n_]^3^/*K*_3_, [N]=[(S)_3_](*t*−*τ*_*N*_) and [P]=*K*_I_^2^/(*K*_I_^2^+[(S)_3_(*t*−*τ*_*P*_)]^2^). *τ*_*P*_ and *τ*_*N*_ represent the time-delays incorporated in the positive and negative feedback loops respectively. Total PSMAD concentration $[\hat {\mathrm {S}}]$ is defined as:

$$[{\hat{\mathrm{S}}}] + \frac{Vn}{Vc} \left([\mathrm{S}_{\mathrm{n}}] + [(\mathrm{S})_{3}]\right) $$

where Vn and Vc are defined as the volume of the nucleus and the cytoplasm compartment, respectively. [(S)_3_]=[S_n_]^3^/*K*_3_ and [S_n_], is calculated from the final equation of 4.

The parameters ${k_{1}^{\text {f-}}}$, ${k_{\text {RC}}^{\text {f-}}}$ and ${k_{\text {PC}}^{\text {f-}}}$ represent, respectively, the strength of the negative feedback on R, RC and *PC*, the R1-associated membrane complexes. Although we have applied the negative feedback on R, RC and *PC* simultaneously and with identical strengths and binding constants, the feedback on *PC* is what produces the switching behaviour (see “Site of negative feedback for TGF-*β* signalling” section). The positive feedback is applied only to R, where the polyamines act [26, 29]. The cooperativity of the RF - TGF-*β* signalling system originates from the coupling of the self-regulatory positive and negative feedback rather than from extracellular effects such as ligand dimerization or depletion.

The component P in Eq. 4 represents the Azin1: Antizyme:ODC:Polyamine associated reactions through which the positive feedback acts on the receptors (Fig. 1). The positive feedback is indirect, being affected by two coupled, inhibitory processes [26].

To achieve the most biologically compatible and robust model of TGF-*β* signalling, the sites of action of the feedback reactions needs to be determined. Sensitivity analysis identified PC as the negative feedback action point (see “Site of negative feedback for TGF-*β* signalling” section). SMAD7 binds to receptors and participates in the induction of E3 ubiquitin (Ub) ligase-mediated receptor ubiquitination [63, 64]. Henri-Michaelis-Menten kinetics is used to model the negative feedback inhibitory function. It is been reported that polyamine depletion increases the TGF-*β* type 1 receptor mRNA and increases the sensitivity of cells to TGF-*β*- mediated growth inhibition [26, 28, 29]. Consequently, we have modelled successive reactions of the positive feedback loop using two inhibitory reactions: first, the inhibition the intermediate inhibitor P via miR-433 and second, the inhibition of R via P.

Time delays are required in the reactions initiated by (S)_3_. Hence, time-delays have been applied to both the positive and negative feedback loops. The time-delays compensate for the SMAD nucleocytoplasmic shuttling and the other reactions that have been consolidated in the reduced models (e.g. SMAD7 transcription and translation for the negative feedback loop and the miR-433/Azin1/Antizyme/ODC reaction chain for the positive feedback loop).

Simulations described in the results were performed with the equations described in the RF- model. Concentrations are dimensionless and scaled such that *v*_1_=1. More simulation and experiment results are shown in section “Supplementary Figures” of “Additional file 1”.

## Results and discussion

### Numerical simulations of TGF-*β* signalling

Analyses of the reduced equations and scaling make it possible to study the characteristics of the model with less complexity. Our model uses six coupled differential equations to represent all the reactions occurring on the membrane, within the SMAD signalling cascade and during the feedback loops. In all the computer simulations we have assumed *τ*_*P*_=*τ*_*N*_=45 minutes.

In order to ensure the existence and uniqueness of the solution (or the steady-state), the system must satisfy the global/local Lipschitz condition [65]. All the equations defined by Eq. 4 can be considered in the form of state equations, $\dot {x} = f(x,t)$, and are globally continuous in x and t. Also their partial derivatives $\left (\frac {\partial f_{i}}{\partial x_{j}}\right)$ are continuous for all x ∈*R* ^*n*^, n =6. Since the partial derivatives $\left (\frac {\partial f_{i}}{\partial x_{j}}\right)$ are locally bounded, it can be inferred that all f _*i*_(x,t) are locally Lipschitz for all x. Therefore, the state equations in Eq. 4 ensure the existence of a unique solution in the domain of interest.

Note that the domain of interest D, where x ∈D, is a subset of R ^6^. Several biological constraints are applied to the model parameters and the initial values of the variables. For instance, none of the components of the model can be negative nor infinite since they are concentrations, kinetic rates or binding constants. Many of the cytoplasmic and nuclear variables are zero at the beginning of the stimulation. Consequently, D does not cover entire R ^6^ space.

**Fig. 4:**
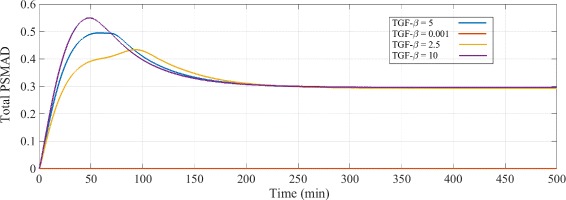
Total PSMAD time-course for different TGF-*β* concentrations. The TGF-*β* signalling and hence the PSMAD time-course is proportional to the TGF-*β* concentration. As the TGF-*β* input signal increases, the peak of the total PSMAD concentration is shifted to the left, is stronger and lasts longer. In the case of approximately zero TGF-*β* input (TGF-*β*=0.001), the signalling does not occur. Despite the short-term changes in the total PSMAD concentration (< 3 hours) with respect to TGF-*β*, its steady-state level remains the same (0.3). We have parametrize our RF- model based on its consistency with the experimental data, i.e. TGF-*β*=5, where the peak in the total PSMAD concentration 50-60 min after the stimulation corresponds to the short-term (transient) response and the constant level at 0.3 represents the long-term (steady-state) response of the system. Note that all concentrations are represented with arbitrary units

To test our hypothesis that the positive feedback is responsible for the change of the behaviour of the system for both short-term (0-3 hours) and long-term (6-8 hours) cellular responses, we ran the simulations for the same TGF-*β* concentration and for stimulation times up to 8 hours. The parameter values used to populate the RF- TGF-*β* model equations are shown in “Additional file 1: Table S3”. Figure 4 shows the predicted changes in the PSMAD concentration time-course for different TGF-*β* concentrations. Despite noticeable changes in the transient response of the model to different (non-zero) ligand concentrations, the steady-state remains unchanged. Zero TGF-*β* input initiates no signalling, as we expected. In order to reproduce the results of the total PSMAD time-course in the literature (e.g. [22, 66]), we have parametrized the RF- model with TGF-*β*=5 arbitrary unit, where the steady-state level of PSMAD is 40*%* less than its short-term peak value and peaks one hour after the ligand stimulation.

The RF- model of TGF-*β* signalling can show oscillations under certain conditions (Additional file 2: Figure S5). Oscillation occurs because of the coupling between the positive and negative feedback loops. More specifically, increasing the receptor production rate (*v*_1_) and SMAD production rate (*v*_S_) at the same time increases the potential components which are necessary for signalling when the ligand is abundant. Therefore, the system can oscillate without decaying of the PSMAD levels. While the model can produce oscillatory responses, no oscillation has been reported in TGF-*β* signalling pathway experimentally. As a result, we adjusted the RF- model parameters and kinetic rates such that PSMAD experiences a single peak after the stimulation and decays smoothly to the steady-state level.

The Zi et al. model [22] produced a sigmoidal TGF-*β* concentration dependence for the cellular responses to long-term stimulation. The total concentration of PSMAD was used as an interpretation of the final cellular response. According to their results [22], the Hill coefficient of the fitted curve to the cell responses to long-time TGF-*β* stimulation was approximately 4.5. The Zi et al. model’s short-term (transient) responses to TGF-*β* followed the Hill equation with an approximate coefficient of 0.8 [22]. Zi et al. proposed that the reason for such a dramatic change in the behaviour of the system was due to a significant time-dependent ligand depletion caused ligand-receptor interaction and consequential degradation of the ligand [22].

**Fig. 5:**
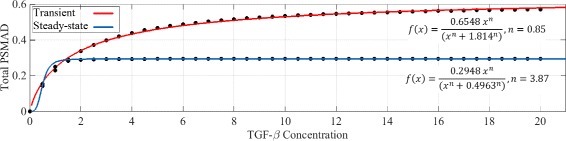
Transient and steady-state responses of the simplified TGF-*β* signalling model. Short-term responses of PSMAD levels to different concentrations of TGF-*β* is referred as transient response. The simulation time for each point in this figure is 50 min (the time of overshoot in Fig. 4). Long-term responses of PSMAD levels to different concentrations of TGF-*β* is referred as steady-state response. The simulation time for each point in this figure is 500 min (the time of steady-state in Fig. 4). The only parameter of the model which is being changed in producing both curves is the TGF-*β* concentration. Note that the unit of concentration on both axes are arbitrary

We examined the short- (0-3 hours) and long- (6-8 hours) term responses for PSMAD in our model as a function of TGF-*β* concentration (Fig. 5). The Hill coefficients are 0.85 for the short-term and 3.87 for the long-term stimulation, i.e. similar to the values determined by Zi et al. (see Zi’s Figure 5.A and 5.B [22]). The parameter values are fitted to a single term (Hill coefficient) in Fig. 5 and Additional file 2: Figure S3. Note that the dots are the results of the RF- model simulation and the curve show the fitted Hill equation. These results support our hypothesis that the coupling of time-delayed positive and negative feedbacks in the TGF-*β* signal transduction system can account for ultra-sensitive responses to the ligand concentrations.

### Site of negative feedback for TGF-*β* signalling

In an initial calculation we allowed the negative feedback to operate on all of the R1-associated complexes on the membrane, however, sensitivity analysis indicated that it is the negative feedback through PC which regulates the system. PC is the only TGF-*β*-associated complex in the simplified model for TGF-*β* signalling. The total TGF-*β* ligand concentration (extracellular TGF-*β*, which is kept constant in our simulations, and that which is bound within the *PC* complex) decreases because of the degradation of *PC* via the basal degradation of, and negative feedback on, *PC*. The saturation of the system with TGF-*β* flattens the TGF-*β* concentration response curves at high concentrations of ligand (Fig. 5). In order to examine our hypothesis, we conducted a set of simulations with the feedback on R and RC removed (Figure S3). To accomplish this, $k_{1}^{\text {f}-}$ and $k_{\text {RC}}^{\text {f}-}$ were set to zero. The results of these simulations corroborated our initial hypothesis that the negative feedback acts almost entirely through PC.

The dynamics and the effect of the feedback loops depend on other parameters i.e. N and K. However, other parameters cannot be set to zero, as these concentrations, e.g. N, depend on other concentrations in the system, such as (S)_3_, which is non-zero after the initial time point. Consequently, N is not zero after time 0. K is the binding constant of the reaction and is in the denominator together with another concentration, e.g. R in Eq. 4. Setting K large enough does not guarantee that the negative feedback loop will be turned off. Setting coefficients to zero is the only way of removing the effect of a negative feedback loop from components R and RC. The negative feedback loop is only acting on R, RC and PC. If we remove its effect on R and RC, PC is the only component that is affected and regulated by negative feedback loop. Please note that turning off the negative feedback loop for one component does not alter the effectiveness of this loop on the other components: N is considered as an enzyme in the equations (Michaelis -Menten kinetics) and is not consumed during the reactions, so its concentration and hence its effectiveness does not change.

### Cancer cells: changes in response to TGF-*β*

**Fig. 6:**
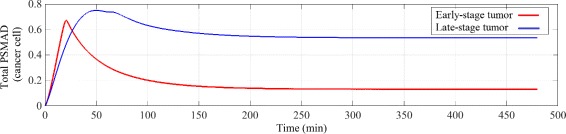
Total PSMAD time-course for a certain TGF-*β* concentration. Total PSMAD time-course for a certain TGF-*β* concentration. Simulation results for low membrane receptor concentration condition (or so called early-stage tumors) are compared with the simulation results for high membrane receptor concentration condition (or so called late-stage tumors). These conditions were simulated via altering the receptor production rate on the membrane. Note that the units of PSMAD concentration levels are arbitrary

We propose that the time-course of the PSMAD concentration in response to TGF-*β* stimulation is modified in cancer cells due to the possible mutations in SMADs, mutations to TGF-*β* receptors and/or different receptor levels [67–70]. Consequently, we simulated the biochemical conditions of the early-stage tumors by reducing the TGF-*β* receptor levels and the SMAD concentrations [71]. More precisely for modulating the receptor levels, we decreased the effect of the positive feedback loop on the receptors ($k_{1}^{\text {f}+}$ in Additional file 1: Table S3 is decreased from 1 to 0.1) and SMADs (*v*_*s*_ in Additional file 1: Table S3 is decreased from 1 to 0.5). The simulation response of the total PSMAD time-course in cells with lower receptor and SMAD concentrations is plotted in Fig. 6. A comparison of Fig. 6 with Fig. 4 reveals that PSMAD concentration peaks to a higher level (0.67 rather than 0.5) but reduces to a lower level at the steady-state (0.13 v.s. 0.3). Clearly at lower receptor levels (< 0.5 normal), e.g. found in early cancer, the responses to TGF-*β* are reduced significantly. This result confirms the suitability of our simplified receptor model of TGF-*β* signalling for simulating the responses in both normal cells and the early colon cancer cells.

In contrast, late-stage tumors are more responsive to TGF-*β* signalling [72]. This could be due to the effects of TGF-*β* on the micro-environment and consequential indirect stimulation of the tumor [73–77]. However, where the TGF-*β* receptor is intact and SMADs are mutated, active receptors and signalling via the MAPK and P38 pathways can stimulate migration and invasion [41, 73, 78]. In order to simulate late tumor environment, the receptors and SMADs levels are increased, by increasing the relative kinetic rates. *v*_1_ in Additional file 1: Table S3 is increased from 1 to 1.2 and *v*_S_ is increased from 1 to 1.5. The predicted responses of late-stage tumors to TGF-*β* stimulation are shown in Fig. 6. Although total PSMAD concentration peaks at a higher level of TGF-*β* receptor in late tumors, the steady-state levels of PSMAD are not significantly different from the peak (i.e. normal levels of TGF-*β* receptor).

**Fig. 7:**
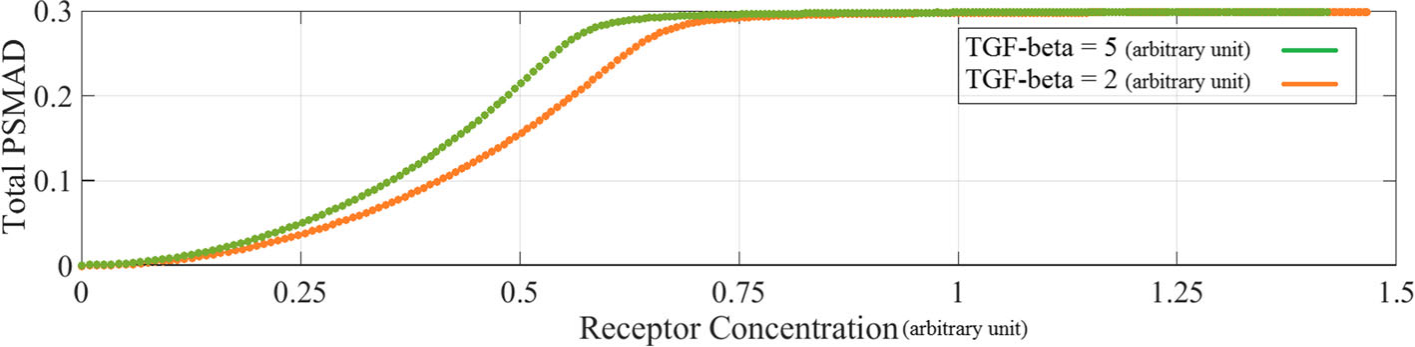
The effects of receptor concentration on the long-term response of PSMAD (500 min). The PSMAD steady-state levels are calculated for two distinct ligand concentrations TGF-*β* = 2 and TGF-*β* = 5 (arbitrary units). Approximately, no difference is observed between the two curves of this figure. Note that the units of PSMAD and receptor concentration levels are arbitrary

To investigate the role of receptor level in the signalling, we have simulated the behaviour of PSMAD concentration while the receptor concentration increases monotonically. Receptor production rate was increased to achieve an increase in receptor concentration. TGF-*β* concentration was maintained at a constant level during the experiment. This simulation was conducted for two distinct concentrations of TGF-*β*: 5 and 2 (arbitrary units). The second TGF-*β* concentration is located approximately where the switch in the long-term steady-state PSMAD concentration occurs (see steady-state responses in Fig. 5 and Additional file 2: Figure S3). There was no distinguishable change in the PSMAD steady-state concentration when TGF-*β* concentration was reduced (Fig. 7). Low receptor concentrations simulate cancer cells (see Fig. 7). The non-responsiveness at the start in both panels of Fig. 7 show that the cells are insensitive to TGF-*β* signalling when the receptor copy numbers are very low, i.e. the situation in cancer cells. The saturation level determines the receptor concentration in which the highest level of signal occurs. When receptor concentration is approximately 0.75, the PSMAD level reaches a saturation level and stays there as the receptor concentration increases. The saturation levels in Fig. 7 correspond to the steady-state of PSMAD in Fig. 5, steady-state response. As expected, when the TGF-*β* concentration is increased the curve of PSMAD shift to the left and all the changes happen at lower receptor levels.

According to the RF- model formulation, the negative feedback term is directly proportional to −((S)_3_)^2^, while the positive feedback term changes in proportion to $-\frac {1}{(\text {(S)}_{\text {3}})^{2}}$. As a result, negative feedback dominates the positive feedback at high (S)_3_ concentrations (e.g. at the peak value of PSMAD) and decreases the PSMAD level until it reaches a stable state (see Fig. 7 and section “Feedback Loops and Time-Delays in the RF- Model” in “Additional file 1”).

**Fig. 8:**
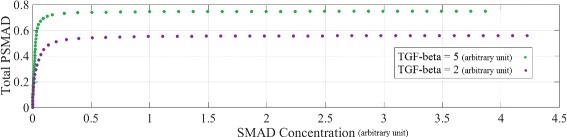
The effects of SMAD concentration on the long-term response of PSMAD (500-2500 min). The PSMAD steady-state levels are calculated for two distinct ligand concentrations TGF-*β* = 2 and TGF-*β* = 5 (arbitrary units). The steady-state level of total PSMAD rises higher when TGF-*β*=5 than when TGF-*β*=2 due to the increase in the ligand concentration. Note that the units of PSMAD and SMAD concentration levels are arbitrary

The results of the simulations with different initial SMAD concentrations are shown in Fig. 8. The PSMAD levels in these simulations are sensitive to the TGF-*β* concentration. As expected the PSMAD levels increase with the SMAD levels until they saturate. Decreasing the TGF-*β* value suppressed the signal at all SMAD concentrations. At the higher concentration of TGF-*β*, PSMAD levels reach the saturation level at lower SMAD concentration i.e. 0.1 (Fig. 8, TGF-*β*=5) compared to 0.4 in Fig. 8 when TGF-*β*=2. The difference in the saturation levels of the two curves in Fig. 8 is due to the different steady-state levels of PSMAD time-course, stimulated by different TGF-*β* concentrations. Furthermore, as the initial concentration of SMAD increases, the RF- model reaches its steady-state later (due to damped oscillation of PSMAD level, Additional file 2: Figure S5).

## Comparison of simulation results with experimental data

**Fig. 9:**
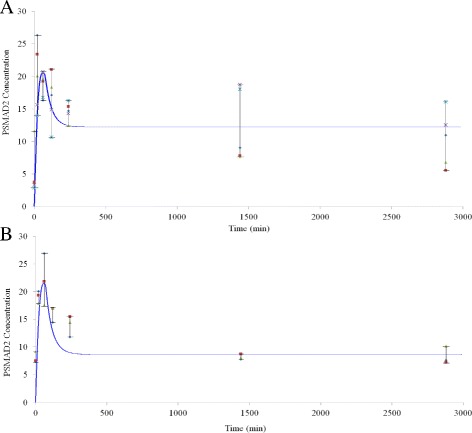
PSMAD2 time-course validation with experimental data sets from **a** wild type and **b** Gp13 0^*F*/*F*^ MEFs. Different colors of dots specify different experiments. The curves represent the model prediction of PSMAD2 dynamics. The model parameters are changed in the curve of Fig. 9 b so that the steady-state level of PSMAD2 concentration is lower and its peak is higher

Our simplified TGF-*β* signalling RF- model was tested experimentally using PSMAD data from mouse embryonic fibroblasts. The predicted results from the model are compared to two different experimental data sets in Fig. 9. The difference between the experimental data and the simulation curves can be explained by the errors associated with the experiments and lack of experimental data to parameterize the model. The simulation results are in good agreement with the experimental results from the response to TGF-*β* signalling in normal cells (Fig. 9 a and b).

The experimental data from wild type MEFs and the model prediction curve for total PSMAD2 concentration level are plotted in Fig. 9 a. Similarly, in Fig. 9 b the simplified model is plotted with the experimental data set from Gp13 0^*F*/*F*^ MEFs [53]. In order to achieve the best fit in Fig. 9 b the parameters of the RF- model had to be adjusted. It has been reported that the level of the SMAD7 concentration is higher in Gp13 0^*F*/*F*^ MEFs due to their gene modification [53]. As is shown in Fig. 9 b, the steady-state level of PSMAD2 is lower than in Fig. 9 a. Note that the error bars are smaller in Fig. 9 b for the longer time points.

## Conclusions

The importance of TGF-*β* signalling in the progression of cancer heralded in a new era of cancer cell biology research [73, 79–81]. Several models for TGF-*β* signalling have now been proposed [16–22]. In each case these models attempted to study the responses of the intracellular signalling reactions to different concentrations of TGF-*β*. In one of the most comprehensive mathematical models Zi et al. [22] predicted that ligand depletion contributed to the long-term response levels of PSMAD. Zi et al. suggested that at higher concentrations of TGF-*β*, there was no depletion from the medium and as a result there was a transfer from a transient to a switch-like response to the TGF-*β* concentration. However, they also noted the possibility that negative feedback mechanisms might also contribute to the switch-like response [22].

Our TGF-*β* model uses fewer reactions than Zi et al. [22], however our model represents the behaviour of the critical components that control the responses to TGF-*β* stimulation over both 80 min and 8 hr time-frames. It is known that time-delayed positive and negative coupled feedbacks can create robust stable signalling [32, 33, 82, 83]. In order to explore the critical role of feedback loops in the TGF-*β* signalling networks we introduced a model where the steady-state was dependent on positive and negative feedback loops. One of the objectives of our study was to design a mathematical model that is applicable to both normal cells and cancer cells. In many early cancer cells the number of TGF-*β* receptors decreases significantly [68–70], thus TGF-*β* signalling is down-regulated. The time-dependent ligand depletion model of Zi et al. [22] does not simulate this decrease in the receptor levels.

Our simulation results show that the PSMAD response of the cells is less sensitive to TGF-*β* stimulation at low receptor concentration. This is consistent with TGF-*β* signal suppression in early cancer cell lines. Our simulations also indicate that reduction in SMAD levels will also cause a global suppression of signalling in response to TGF-*β*. Due to mutations of SMADs, many early cancers are likely to have reduced levels of TGF-*β* signalling [67]. These results are consistent with the picture of early-stage tumors being associated with the loss of TGF-*β* sensitivity and the decrease of TGF-*β* receptor expression [84].

TGF-*β* signal transduction can be stimulated in late-stage tumors (“The TGF-*β* Paradox” [85, 86] i.e. early-stage cancers are less sensitive to TGF-*β* inhibitor, whereas many late-stage cancers are stimulated by TGF-*β*, either directly through increased receptor levels or indirectly by effects on the micro-environment of the cells). Our model with feedback loops produces results consistent with both roles of TGF-*β* in tumorigenesis. In the late-stage tumors the increased responsiveness to TGF-*β* could occur via increased production rates for receptors or SMADs. According to our model predictions, the overshoot peak of PSMAD in response to TGF-*β* is higher in early tumors and the steady-state levels of PSMAD are lower generally, while in late tumors both steady-state and peak levels are higher than normal cells. Additionally, the difference between the PSMAD peak and steady-state levels is less in late-stage tumors, so the TGF-*β* signalling would be on for longer times. This work can be used as a guide for future experimental research on TGF-*β* effects on tumor progression. It must be emphasized that the late-stage tumour responses must be influenced by other genetic changes which change the response to TGF-*β* from inhibition to stimulation. In future studies it will be important to add other pathways which can link the TGF-*β* signalling to anti-mitotic processes, migration processes or even increases proliferation.

This model provides the basis for predicting the effects of TGF-*β* on the signalling processes in cells with different levels of TGF-*β* receptors or SMADs. By considering of a model where coupled, positive-negative feedback loops modulate TGF-*β* signalling switching responses can be observed without depletion of TGF-*β* [22]. TGF-*β* signal transduction can be studied more precisely using control theory analysis including system identification methods [87, 88].

## Methods

The experimental data set and the kinetic rates used to set the initial parameters for the model were taken from the literature [16–22]. For the initial conditions, estimation of parameter values and the interpretation of some experimental data, we have benefitted from the model proposed by Zi et al. [22]. The values for all parameters are documented in the Additional file 1: Tables S1, S2 and S3.

### Computer modelling and simulations

The programs used for these simulation where PYTHON 2.7 and MATLAB 7.10. The curve fitting tool box of MATLAB is used for fitting the Hill equation in Fig. 5 and Additional file 2: Figure S3, and deriving the Hill coefficients.

### Mathematical and biochemical analysis

The biochemical kinetics, equilibrium analysis, feedback analysis, reduction analysis using the rapid equilibrium assumption, time-delayed analysis, asymptotic expansions and sensitivity analysis (refer to “Additional file 1”) have been performed on the model [49].

We have used Western blot analysis for our quantitation. Western blot is only a semi-quantitative method; absolute values are not measured. As a result, throughout the manuscript and Figures, the units for protein concentrations are arbitrary. N.B. each species in Eq. 4 is calculated in its compartment volume. The volume corrections for all species of Eq. 4 are hidden in the coefficients of the corresponding terms and are not explicitly shown in the equations, e.g. ${k_{n}^{-}}$ and ${k_{n}^{+}}$ include the *V*_*n*_/*V*_*c*_ and *V*_*c*_/*V*_*n*_ volume correction terms, respectively.

### Cell culture and cell lysis

Mouse embryonic fibroblasts (MEFs) cells were isolated from day 13 to 15 embryos. MEF WT, SV40-immortalized MEFs (Simian vacuolating virus 40) and Gp130 ^*F*/*F*^ MEFs [53] were cultured in DMEM containing 15% FCS. The cells were typsinazed and washed with DMEM + 15% FCS before plating. Passage 3 cells with 1 ×10^6^ MEFs/well were seeded in 60 mm plates for 0-4 hours, 0.5 ×10^6^ MEFs/well for 24 hour and 0.25 ×10^6^ MEFs/well for 48 hour treatment with 5 ng/ml TGF-*β* respectively. After washing with cold PBS for two times, cells were lysed in ice-cold 200 *μ*l RIPA lysis buffer, containing 1M Tris/HCL, 0.5 M EDTA, 5M NaCl, 10% Na Doc (Sodium Deoxycholate), 10% TX-100, 10% SDS, proteinase inhibitor 100 × and H _2_O. The cell lysates were passed through 27 G needle 5 times, then incubated on ice for 20 min. After incubation the samples were spun at 13,000 rpm for 30 min at 4 ^*o*^C. The supernatant was transferred to new tubes: 20 *μ*l of samples used for the BCA protein assay (Sigma kit B9643); 20 *μ*l 5 × sample buffer was added to 80 *μ*l of sample, the samples heated at 95 ^*o*^C for 10 min and analysed by SDS-PAGE.

### Western blotting

Novex NuPAGE *Ⓡ* 4-12%-Bis-Tris (life technologies NP0335 Box) gels were used to analyse the sample lysates from each time point. The SMAD7 antibody was provided via Santa Cruz Biotechnology and was used at 1:1000 in 3% BSA-TBS-T. PSMAD2 antibody (rabbit polyclonal anti-phospho-Smad2 antibody (1:1000 for Western blot)) was a gift from Prof. Peter ten Dijke (Leiden University Medical Center, Netherlands). *β*-tubulin, actin, Lamin B1 or transferrin receptor were used as loading controls depending on the protein being analysed. For antibody detection, the proteins were transferred onto a nitrocellulose membrane using the iBlot 2 gel transfer device (Life technologies) and the membranes were scanned using the Odyssey infrared scanner (LI-COR).

### Protein quantitation

The Western blot images were quantitated using ImageJ 1.49p. The signals from each protein were normalised using the signal from each loading control.

## Additional files

Additional file 1 Mathematical Model for TGF-*β* Signalling, which provides more information about the model design and model reduction steps. Feedback Loops and Time-Delays in the RF- Model, which provides more information about the delayed positive and negative feedback loops and their effects on the signalling system. Parameters for TGF-*β* Signalling Model, which provides tables of parameters involved in the signalling model [89, 90]. (PDF 152 kb)

Additional file 2 Which provides information about extra figures (**Figures S1-S7**) of model simulation and experimental data that help understanding the signalling system. **Figure S1** Dynamics of feedback loops in RF- model. A) The negative feedback loop time-course for a time-delay *τ*_*N*_=20 minutes B) The positive feedback loop time-course for a time-delay *τ*_*P*_=120 minutes. N changes proportionally with (S)_3_ as ((S)_3_) ^2^ while P is inversely proportional to (S)_3_ as 1/(1+((S)_3_)^2^). **Figure S2** The effects of feedback loops on the TGF-*β*receptor concentration dynamics. The time-delays are either for both *τ*_*N*_ and *τ*_*P*_=45 minutes or *τ*_*P*_=120 and *τ*_*N*_=20 minutes. The effects of individual feedback loops on the receptor levels are studied. The peaks and the valleys are due to positive and negative feedback loops respectively. The time-delays shift the peaks and valleys in time. The strength of the feedback loops change the amplitude of the peaks and valleys. **Figure S3** The predicted effects of different concentrations of TGF-*β* on PSMAD levels when the negative feedback loop influences PC only. These effects are shown for the short-term (50 min) and long-term (500 min) responses of the TGF-*β*signalling system. Note that the dots are derived from model simulation and the curves show the Hill equations which are fitted by MATLAB. **Figure S4** PSMAD responses to changes in TGF-*β* concentration at different simulation times. The Hill coefficient increases with the increase in the simulation time. The PSMAD response does not switch before 200 min, where the PSMAD level starts saturating (Fig. 4). The curves of 50 min and 500 min simulation times correspond to the curves in Fig. 5. **Figure S5** PSMAD time-course for different production rates of SMAD The SMAD production rate (*v*_S_) determines the steady-state of the PSMAD response of RF- model. Higher *v*_S_ SMAD concentration during the signalling. The PSMAD time-course experiences damped oscillation for high *v*_S_. The oscillations appear to delay the system reaching its steady-state. **Figure S6** The representative Western blots for PSMAD2 analysed in Fig. 9. A)wild type MEFs B)Gp130 ^*F*/*F*^ MEFs. In each panel, the top bands show the PSMAD2 signals of the double-stimulation experiment. The bottom bands show the actin signals for loading control to which the PSMAD2 levels are normalised to produce the results shown in Fig. 9. **Figure S7** The validation of the simplified model with experimental data. The dots show the level of PSMAD2 concentration obtained from experiment and the curves specify the model predictions. A) PSMAD2 time-course for 0-1h on SV40-immortalised MEFs stimulated with TGF-*β* and its corresponding blot B) PSMAD2 time-course for 0-4h on SV40-immortalised MEFs stimulated with TGF-*β* and its corresponding blot C) PSMAD2 time-course for 0-4h on wild type MEFs stimulated with TGF-*β* and its corresponding blot. (PDF 1750 kb)

## References

[1] Massagué J (1990). The transforming growth factor- *β* family. Annu Rev Cell Biol.

[2] Clarke DC, Liu X (2008). Decoding the quantitative nature of TGF- *β*/Smad signaling. Trends Cell Biol.

[3] Shi Y, Massagué J (2003). Mechanisms of TGF- *β* signaling from cell membrane to the nucleus. Cell.

[4] Feng XH, Derynck R (2005). Specificity and versatility in TGF- *β* signaling through Smads. Annu Rev Cell Dev Biol.

[5] Jenkins G (2008). The role of proteases in transforming growth factor- *β* activation. Int J Biochem Cell Biol.

[6] Massagué J, Seoane J, Wotton D (2005). Smad transcription factors. Genes Dev.

[7] ten Dijke P, Miyazono K, Heldin CH (2000). Signaling inputs converge on nuclear effectors in TGF- *β* signaling. Trends Biochem Sci.

[8] Massagué J (1998). TGF- *β* signal transduction. Ann Rev Biochem.

[9] Nicolás FJ, Hill CS (2003). Attenuation of the TGF- *β*-Smad signaling pathway in pancreatic tumor cells confers resistance to TGF- *β*-induced growth arrest. Oncogene.

[10] Jenkins BJ, Grail D, Nheu T, Najdovska M, Wang B, Waring P, Inglese M, McLoughlin RM, Jones SA, Topley N, Baumann H, Judd LM, Giraud AS, Boussioutas A, Zhu HJ, Ernst M (2005). Hyperactivation of Stat3 in gp130 mutant mice promotes gastric hyperproliferation and desensitizes TGF- *β* signaling. Nat Med.

[11] Massagué J, Blain SW, Lo RS (2000). TGF- *β* signaling in growth control, cancer, and heritable disorders. Cell.

[12] Bachman KE, Park BH (2005). Duel nature of TGF- *β* signaling: tumor suppressor vs. tumor promoter. Curr Opin Oncol.

[13] Leight JL, Wozniak MA, Chen S, Lynch ML, Chen CS (2012). Matrix rigidity regulates a switch between TGF- *β*1-induced apoptosis and epithelial-mesenchymal transition. Mol Biol Cell.

[14] Ikushima H, Miyazono K (2011). Biology of transforming growth factor- *β* signaling. Curr Pharm Biotechnol.

[15] Attisano L, Wrana JL (2002). Signal transduction by the TGF- *β* superfamily. Sci Signal.

[16] Melke P, Jönsson H, Pardali E, ten Dijke P, Peterson C (2006). A rate equation approach to elucidate the kinetics and robustness of the TGF- *β* pathway. Biophys J.

[17] Vilar JMG, Jansen R, Sander C (2006). Signal Processing in the TGF- *β* Superfamily Ligand-Receptor Network. PLoS Comput Biol.

[18] Clarke DC, Brown ML, Erickson RA, Shi Y, Liu X (2009). Transforming growth factor beta depletion is the primary determinant of Smad signaling kinetics. Mol Cell Biol.

[19] Zi Z, Klipp E (2007). Constraint-Based Modeling and Kinetic Analysis of the Smad Dependent TGF- *β* Signaling Pathway. PLoS ONE.

[20] Schmierer B, Tournier AL, Bates PA, Hill CS (2008). Mathematical modeling identifies Smad nucleocytoplasmic shuttling as a dynamic signal-interpreting system. Proc Natl Acad Sci.

[21] Chung SW, Miles FL, Sikes RA, Cooper CR, Farach-Carson MC, Ogunnaike BA (2009). Quantitative modeling and analysis of the transforming growth factor beta signaling pathway. Biophys J.

[22] Zi Z, Feng Z, Chapnick DA, Dahl M, Deng D, Klipp E, Moustakas A, Liu X (2011). Quantitative analysis of transient and sustained transforming growth factor- *β* signaling dynamics. Mol Syst Biol.

[23] Zi Z, Klipp E (2006). SBML-PET: a Systems Biology Markup Language-based parameter estimation tool. Bioinformatics.

[24] Bachmann J, Raue A, Schilling M, Becker V, Timmer J, Klingmuller U (2012). Predictive mathematical models of cancer signalling pathways. J Intern Med.

[25] Nakao A, Imamura T, Souchelnytskyi S, Kawabata M, Ishisaki A, Oeda E, Tamaki K, Hanai J, Heldin CH, Miyazono K, ten Dijke P (1997). TGF- *β* receptor-mediated signalling through Smad2, Smad3 and Smad4. EMBO J.

[26] Li R, Chung AC, Dong Y, Yang W, Zhong X, Lan HY (2013). The microRNA miR-433 promotes renal fibrosis by amplifying the TGF- *β*/Smad3-Azin1 pathway. Kidney Int.

[27] Kahana C (2009). Regulation of cellular polyamine levels and cellular proliferation by antizyme and antizyme inhibitor. Essays Biochem.

[28] Liu L, Santora R, Rao JN, Guo X, Zou T, Zhang HM, Turner DJ, Wang JY (2003). Activation of TGF- *β*-Smad signaling pathway following polyamine depletion in intestinal epithelial cells. Am J Physiology-Gastrointestinal Liver Physiol.

[29] Rao JN, Li L, Bass BL, Wang JY (2000). Expression of the TGF- *β* receptor gene and sensitivity to growth inhibition following polyamine depletion. Am J Physiology-Cell Physiol.

[30] Patel AR, Li J, Bass BL, Wang JY (1998). Expression of the transforming growth factor- *β* gene during growth inhibition following polyamine depletion. Am J Physiology-Cell Physiol.

[31] Shi F, Zhou P, Wang R. Coupled positive feedback loops regulate the biological behavior. IEEE 2012;169–73.

[32] Ferrell JE, Ha SH (2014). Ultrasensitivity part II: multisite phosphorylation, stoichiometric inhibitors, and positive feedback. Trends Biochem Sci.

[33] Ferrell JE, Ha SH (2014). Ultrasensitivity part I: Michaelian responses and zero-order ultrasensitivity. Trends Biochem Sci.

[34] Mitrophanov AY, Groisman EA (2008). Positive feedback in cellular control systems. Bioessays.

[35] Chang DE, Leung S, Atkinson MR, Reifler A, Forger D, Ninfa AJ (2010). Building biological memory by linking positive feedback loops. Proc Natl Acad Sci.

[36] Kleeff J, Ishiwata T, Maruyama H, Friess H, Truong P, Büchler M, Falb D, Korc M (1999). The TGF- *β* signaling inhibitor Smad7 enhances tumorigenicity in pancreatic cancer. Oncogene.

[37] Wagner J, Keizer J (1994). Effects of rapid buffers on Ca2+ diffusion and Ca2+ oscillations. Biophys J.

[38] Massagué J, Gomis RR (2006). The logic of tgf *β* signaling. FEBS Lett.

[39] Lee MK, Pardoux C, Hall MC, Lee PS, Warburton D, Qing J, Smith SM, Derynck R (2007). Tgf- *β* activates erk map kinase signalling through direct phosphorylation of shca. EMBO J.

[40] Mu Y, Gudey SK, Landström M (2012). Non-smad signaling pathways. Cell Tissue Res.

[41] Wang X, Li X, Ye L, Chen W, Yu X (2013). Smad7 inhibits tgf- *β*1-induced mcp-1 upregulation through a mapk/p38 pathway in rat peritoneal mesothelial cells. Int Urol Nephrol.

[42] Yu L, Hébert MC, Zhang YE (2002). Tgf- *β* receptor-activated p38 map kinase mediates smad-independent tgf- *β* responses. EMBO J.

[43] Wieser R, Wrana J, Massagué J (1995). GS domain mutations that constitutively activate T beta RI, the downstream signaling component in the TGF- *β* receptor complex. EMBO J.

[44] Heldin CH, Miyazono K, Ten Dijke P (1997). TGF- *β* signalling from cell membrane to nucleus through SMAD proteins. Nature.

[45] Hayes S, Chawla A, Corvera S (2002). TGF *β* receptor internalization into EEA1-enriched early endosomes role in signaling to Smad2. J Cell Biol.

[46] Massagué J, Kelly B (1986). Internalization of transforming growth factor- *β* and its receptor in BALB/c 3T3 fibroblasts. J Cell Physiol.

[47] Zhang Y, Feng XH, Derynck R (1998). Smad3 and Smad4 cooperate with c-Jun/c-Fos to mediate TGF- *β*-induced transcription. Nature.

[48] Vincent T, Neve EP, Johnson JR, Kukalev A, Rojo F, Albanell J, Pietras K, Virtanen I, Philipson L, Leopold PL (2009). A SNAIL1–SMAD3/4 transcriptional repressor complex promotes TGF- *β* mediated epithelial–mesenchymal transition. Nat Cell Biol.

[49] Fall CP (2002). Computational Cell Biology Interdisciplinary Applied Mathematics; V. 20.

[50] Luo K, Lodish H (1996). Signaling by chimeric erythropoietin-TGF- *β* receptors: homodimerization of the cytoplasmic domain of the type I TGF- *β* receptor and heterodimerization with the type II receptor are both required for intracellular signal transduction. EMBO J.

[51] Ebner R, Chen RH, Shum L, Lawler S, Zioncheck TF, Lee A, Lopez AR, Derynck R (1993). Cloning of a type I TGF- *β* receptor and its effect on TGF- *β* binding to the type II receptor. Science.

[52] Wu JW, Hu M, Chai J, Seoane J, Huse M, Li C, Rigotti DJ, Kyin S, Muir TW, Fairman R (2001). Crystal structure of a phosphorylated Smad2: Recognition of phosphoserine by the MH2 domain and insights on Smad function in TGF- *β* signaling. Mol Cell.

[53] Jenkins BJ, Grail D, Nheu T, Najdovska M, Wang B, Waring P, Inglese M, McLoughlin RM, Jones SA, Topley N (2005). Hyperactivation of Stat3 in gp130 mutant mice promotes gastric hyperproliferation and desensitizes TGF- *β* signaling. Nat Med.

[54] Budi EH, Xu J, Derynck R. Regulation of TGF- *β* Receptors. Methods in molecular biology (Clifton, NJ). 2016; 1344:1. 10.1007/978-1-4939-2966-5_1 26520115

[55] Asano Y, Ihn H, Yamane K, Kubo M, Tamaki K (2004). Impaired smad7-smurf–mediated negative regulation of tgf- *β* signaling in scleroderma fibroblasts. J Clin Investig.

[56] Kang JS, Liu C, Derynck R (2009). New regulatory mechanisms of TGF- *β* receptor function. Trends Cell Biol.

[57] Massagué J, Attisano L, Wrana JL (1994). The TGF- *β* family and its composite receptors. Trends Cell Biol.

[58] Groppe J, Hinck CS, Samavarchi-Tehrani P, Zubieta C, Schuermann JP, Taylor AB, Schwarz PM, Wrana JL, Hinck AP (2008). Cooperative assembly of TGF- *β* superfamily signaling complexes is mediated by two disparate mechanisms and distinct modes of receptor binding. Mol Cell.

[59] Kingsley DM (1994). The TGF- *β* superfamily: new members, new receptors, and new genetic tests of function in different organisms. Genes Dev.

[60] Gunawardena J (2014). Time-scale separation–michaelis and menten’s old idea, still bearing fruit. FEBS J.

[61] von Gersdorff G, Susztak K, Rezvani F, Bitzer M, Liang D, Böttinger EP (2000). Smad3 and smad4 mediate transcriptional activation of the human smad7 promoter by transforming growth factor *β*. J Biol Chem.

[62] Yan X, Liao H, Cheng M, Shi X, Lin X, Feng XH, Chen YG (2016). Smad7 protein interacts with receptor-regulated smads (r-smads) to inhibit transforming growth factor- *β* (tgf- *β*)/smad signaling. J Biol Chem.

[63] Ebisawa T, Fukuchi M, Murakami G, Chiba T, Tanaka K, Imamura T, Miyazono K (2001). Smurf1 interacts with transforming growth factor- *β* type I receptor through Smad7 and induces receptor degradation. J Biol Chem.

[64] Kavsak P, Rasmussen RK, Causing CG, Bonni S, Zhu H, Thomsen GH, Wrana JL (2000). Smad7 binds to Smurf2 to form an E3 ubiquitin ligase that targets the TGF *β* receptor for degradation. Mol Cell.

[65] Khalil HK (1996). Nonlinear Systems.

[66] Inman GJ, Nicolás FJ, Hill CS (2002). Nucleocytoplasmic shuttling of Smads 2, 3, and 4 permits sensing of TGF- *β* receptor activity. Mol Cell.

[67] Fleming NI, Jorissen RN, Mouradov D, Christie M, Palmieri M, Day F, Li S, Tsui C, Lipton L, Sakthianandeswaren A (2013). SMAD2, SMAD3 and SMAD4 mutations in colorectal cancer. Cancer Res.

[68] Wakefield LM, Smith DM, Masui T, Harris CC, Sporn MB (1987). Distribution and modulation of the cellular receptor for transforming growth factor- *β*. J Cell Biol.

[69] Laiho M, Weis M, Massagué J (1990). Concomitant loss of transforming growth factor (TGF)- *β* receptor types I and II in TGF- *β*-resistant cell mutants implicates both receptor types in signal transduction. J Biol Chem.

[70] Kimchi A, Wang XF, Weinberg RA, Cheifetz S, Massagué J (1988). Absence of TGF- *β* receptors and growth inhibitory responses in retinoblastoma cells. Science.

[71] Yu M, Trobridge P, Wang Y, Kanngurn S, Morris S, Knoblaugh S, Grady W (2014). Inactivation of TGF- *β* signaling and loss of PTEN cooperate to induce colon cancer in vivo. Oncogene.

[72] Liu RY, Zeng Y, Lei Z, Wang L, Yang H, Liu Z, Zhao J, Zhang HT (2014). Jak/stat3 signaling is required for tgf- *β*-induced epithelial-mesenchymal transition in lung cancer cells. Int J Oncol.

[73] Pickup M, Novitskiy S, Moses HL (2013). The roles of TGF [beta] in the tumour microenvironment. Nat Rev Cancer.

[74] Giampieri S, Manning C, Hooper S, Jones L, Hill CS, Sahai E (2009). Localized and reversible tgf *β* signalling switches breast cancer cells from cohesive to single cell motility. Nat Cell Biol.

[75] Langenskiöld M, Holmdahl L, Falk P, Angenete E, Ivarsson ML (2008). Increased tgf-beta1 protein expression in patients with advanced colorectal cancer. J Surg Oncol.

[76] Shariat SF, Shalev M, Menesses-Diaz A, Kim IY, Kattan MW, Wheeler TM, Slawin KM (2001). Preoperative plasma levels of transforming growth factor beta1 (tgf- *β*1) strongly predict progression in patients undergoing radical prostatectomy. J Clin Oncol.

[77] Xiong B, Gong LL, Zhang F, Hu MB, Yuan HY (2002). Tgf beta˜ 1 expression and angiogenesis in colorectal cancer tissue. World J Gastroenterol.

[78] Xu J, Acharya S, Sahin O, Zhang L, Lowery FJ, Sahin AA, Zhang XH-F, Hung MC, Yu D (2015). Abstract lb-202: 14-3-3 *ζ* turns tgf- *β*’s function from tumor suppressor to metastasis promoter in breast cancer by contextual changes of smad partners from p53 to gli2. Cancer Res.

[79] Santibanez JF, Quintanilla M, Bernabeu C (2011). TGF- *β*/TGF- *β* receptor system and its role in physiological and pathological conditions. Clin Sci.

[80] Anzano M, Roberts A, Smith J, Sporn M, De Larco J (1983). Sarcoma growth factor from conditioned medium is composed of both type *α* and type *β* transforming growth factors. Proc Natl Acad Sci U S A.

[81] De Larco JE, Todaro GJ (1978). Growth factors from murine sarcoma virus-transformed cells. Proc Natl Acad Sci.

[82] Wagner J, Ma L, Rice J, Hu W, Levine A, Stolovitzky G (2005). p53–Mdm2 loop controlled by a balance of its feedback strength and effective dampening using ATM and delayed feedback. IEE Proc Syst Biol.

[83] Wagner J, Stolovitzky G (2008). Stability and time-delay modeling of negative feedback loops. Proc IEEE.

[84] Duffy I, Varacallo P, Klerk H, Hawker J (2015). Endothelial and cancer cells have differing amounts of tgf beta receptors involved in angiogenesis. FASEB J.

[85] Hansson GK, Libby P (2006). The immune response in atherosclerosis: a double-edged sword. Nat Rev Immunol.

[86] Akhurst RJ, Derynck R (2001). TGF- *β* signaling in cancer–a double-edged sword. Trends Cell Biol.

[87] Chen BS, Wu CC (2012). On the calculation of signal transduction ability of signaling transduction pathways in intracellular communication: systematic approach. Bioinformatics.

[88] Choi S (2010). Systems Biology Approaches: Solving New Puzzles in a Symphonic Manner. Systems Biology for Signaling Networks.

[89] Kavsak P, Rasmussen RK, Causing CG, Bonni S, Zhu H, Thomsen GH, Wrana JL (2000). Smad7 Binds to Smurf2 to Form an E3 Ubiquitin Ligase that Targets the TGFbeta Receptor for Degradation. Mol Cell.

[90] Di Guglielmo GM, Le Roy C, Goodfellow AF, Wrana JL (2003). Distinct endocytic pathways regulate TGF- *β* receptor signalling and turnover. Nat Cell Biol.

