# Supplementary material for: Mathematical model of TGF-βsignalling: feedback coupling is consistent with signal switching
Source: BMC Syst Biol. 2017 Apr 13;11:48. doi: 10.1186/s12918-017-0421-5 (PMC5390422; doi:10.1186/s12918-017-0421-5)
Supplement: Supplementary file 1 — Mathematical Model for TGF-β Signalling, which provides more information about the model design and model reduction steps. Feedback Loops and Time-Delays in the RF- Model, which provides more information about the delayed positive and negative feedback loops and their effects on the signalling system. Parameters for TGF-β Signalling Model, which provides tables of parameters involved in the signalling model [89, 90]. (PDF 152 kb) [file 12918_2017_421_MOESM1_ESM.pdf]

Supplementary_Material

Mathematical Model for TGF$-\beta$ Signalling

The semi-reduced model is described by equations 2. This model can be considered as two subsystems: the membrane subsystem is represented by the first 7 equations and nucleocytoplasmic subsystem is described by the remaining 4 differential equations.

In the receptor subsystem, it is almost impossible to measure k^+ (the forward reaction rate) in the dimerization reactions. Although k^- (the backward reaction rate) is measurable, the easiest parameter to be quantified is K ($K = \frac{k^-}{k^+}$), i.e. the equilibrium constant. The dimerization process of R1 or R2 is much faster than the other receptor reactions. Furthermore, as discussed before the symbolic feedback loop here is replaced with a number of relatively slow reactions. In comparison to every other reaction in the receptor system, the time scales of R1 and R2 dimerization and RC formation are small enough justify the rapid equilibrium assumption [1, 2, 3].

Based on the above assumptions, we apply the conservation law to R1 and R2 , so the final form of the reduced version of the R1 , R2 and PC are:

$$\begin{aligned} R1_T &= R1 + 2(R1)_2 \\ R2_T &= R2 + 2(R2)_2 \\ PC_T &= LC + PC \end{aligned} \tag{1}$$

The conservation law for R1 and R2 states that the total amount of R1 and R2 , by definition, equals to the total amount of R1 and R2 as monomers adding by twice of the amount of R1 and R2 as dimers (which is twice of R1 and R2). The same definition applies for PC_T.

The dimerization reactions, the binding reactions of the two dimers and the phosphorylation of the two R1 s inside LC (PC association) are relatively fast compared to the ligand binding reaction [2]. In nucleocytoplasmic subsystem, inside the cytosol, the trimerization of the phosphorylated SMAD occurs more rapidly than the SMAD phosphorylation processes. Pursuant to the rapid equilibrium assumption, at equilibrium the forward and the backward terms of the fast reactions can be considered to be equal. Therefore, the equations of the two subsystems are represented by:

$$
\begin{aligned}
\beta_1\,[\mathrm{R1}]\,' &= v_1 - k_1\,[\mathrm{R}] - 2k_{\mathrm{RC}}^{+}\,[\mathrm{R1}]\,[\mathrm{R2}] + 2k_{\mathrm{RC}}^{-}\,[\mathrm{RC}] - k_1^{\mathrm{f}+}\,[\mathrm{P}]\,\frac{[\mathrm{R1}]}{[\mathrm{R1}]+K} - \\
&\quad k_1^{\mathrm{f}-}\,[\mathrm{N}]^2\,\frac{[\mathrm{R1}]}{[\mathrm{R1}]+K} \\
\beta_2\,[\mathrm{R2}]\,' &= v_2 - k_2\,[\mathrm{R2}] - 2k_{\mathrm{RC}}^{+}\,[\mathrm{R1}]\,[\mathrm{R2}] + 2k_{\mathrm{RC}}^{-}\,[\mathrm{RC}] \\
[\mathrm{RC}]\,' &= k_{\mathrm{RC}}^{+}\,[\mathrm{R1}]\,[\mathrm{R2}] - k_{\mathrm{RC}}^{-}\,[\mathrm{RC}] - k_{\mathrm{RC}}\,[\mathrm{RC}] - k_{\mathrm{PC}}^{+}\,[(\mathrm{TGF}{-}\beta)_2]\,[\mathrm{RC}] + \\
&\quad k_{\mathrm{PC}}^{-}\,[\mathrm{PC}] - k_{\mathrm{RC}}^{\mathrm{f}-}\,[\mathrm{S_n}]^{\,2}\frac{[\mathrm{RC}]}{[\mathrm{RC}]+K} \qquad (2) \\
(1+K_{\mathrm{PC}})\,[\mathrm{PC}]\,' &= k_{\mathrm{PC}}^{+}\,[(\mathrm{TGF}{-}\beta)_2]\,[\mathrm{RC}] - k_{\mathrm{PC}}^{-}\,[\mathrm{PC}] - k_{\mathrm{PC}}^{-}\,[\mathrm{PC}] - k_{\mathrm{PC}}^{\mathrm{f}-}\,[\mathrm{N}]^2\,\frac{[\mathrm{PC}]}{[\mathrm{PC}]+K} \\
[\mathrm{S}]\,' &= v_{\mathrm{S}} - k_{\mathrm{S}}\,[\mathrm{S}] - k_{\mathrm{S}}^{+}\,[\mathrm{PC}]\,\frac{[\mathrm{S}]}{[\mathrm{S}]+K_{\mathrm{S}}} + k_{\mathrm{S}}^{-}\,[\hat{\mathrm{S}}] \\
[\hat{\mathrm{S}}]' &= k_{\mathrm{S}}^{+}\,[\mathrm{PC}]\,\frac{[\mathrm{S}]}{[\mathrm{S}]+K_{\mathrm{S}}} - k_{\mathrm{S}}^{-}\,[\hat{\mathrm{S}}] - k_{\hat{\mathrm{S}}}\,[\hat{\mathrm{S}}] - 3k_3^{+}\,[\hat{\mathrm{S}}]^3 + 3k_3^{-}\,[(\mathrm{S})_3]
\end{aligned}
$$

,where

$$
\begin{aligned}
\beta_1 &= 1 + 4\frac{[\mathrm{R1}]}{K_1} \qquad (3) \\
\beta_2 &= 1 + 4\frac{[\mathrm{R2}]}{K_2}
\end{aligned}
$$

and again, $[\mathrm{N}] = [(\mathrm{S})_3]\,(t - \tau_N)$ and $[\mathrm{P}] = K_{\mathrm{I}}{}^2/(K_{\mathrm{I}}{}^2 + [(\mathrm{S})_3\,(\mathrm{t} - \tau_{\mathrm{P}})]^2)$. Note that the equilibrium equations that formulate the reduced model can be written as:

$$
\begin{aligned}
[(\mathrm{R1})_2] &= \frac{[\mathrm{R1}]^{\,2}}{K_1} \qquad [\mathrm{LC}] = K_{\mathrm{PC}}\,[\mathrm{PC}] \\
[(\mathrm{R2})_2] &= \frac{[\mathrm{R2}]^{\,2}}{K_2} \qquad [(\mathrm{S})_3] = \frac{[\hat{\mathrm{S}}]^3}{K_3} = \frac{1}{K_3}\left(\frac{[\mathrm{LC}]\,k_{\mathrm{S}}^{+}\,[\mathrm{S}]}{K_{\mathrm{PC}}\,k_{\mathrm{S}}^{-}\,([\mathrm{S}]+K_{\mathrm{S}})}\right)^3
\end{aligned}
$$

In RF- model, we have assumed that K_1 and K_2 are large and thus, $\beta_1 = \beta_2 = 1$.

Feedback Loops and Time-Delays in the RF- Model

In order to investigate the effects of time-delayed positive and negative feedback loops on the TGF$-\beta$ signalling in RF- model, we have studied the dynamics of S_7 (Figure S1.A) and miR-433 (Figure S1.B). The time-delays incorporated in the feedback loops are chosen differently in Figure S1 (Supplementary Figures), $\tau_N = 20$ and $\tau_P = 120$ minutes. Note that these are different from the adjusted time-delays used to produce model predictions in section "Results and Discussion" ($\tau_N = \tau_P =$ 45 minutes). The negative feedback loop ("N") changes proportional to $((\mathrm{S})_3)^2$ therefore it peaks after remaining zero for 20 minutes and decreases to reach its steady-state level. However, the dynamic of the positive feedback loop ("P") is inversely proportional to $1{+}((\mathrm{S})_3)^2$ and is initiated at 1.

Both N and P act as degradation terms (with negative signs in equations 4), however, P changes inversely with signalling and hence decrease the degradation rate

of R. Figure S2 specifies the effects of the feedback loops and their time-delays individually on the receptor dynamics.

The time-delays shift the position of the peak associated with the positive feedback loop and the valley associated with the negative feedback loop. The height and depth of peaks and valleys are proportional to the amplitude of the feedback loops. In the absence of the feedback loops the steady-state level of the receptors is determined by the production and basal degradation rates ("Without N or P" plot of Figure S2). When negative feedback only is applied to the system, the steady-state does not change significantly, though the receptor level decreases after τ_N minutes. This inhibition cause the signalling to be down-regulated and there is a consequential decrease in the level of S_7 (or N) and the receptor concentration restores rapidly. In the presence of the positive feedback loop, the receptor level rises approximately 50 minutes after TGF$-\beta$ stimulation and slightly decreases due to the balance between the production and degradation terms of the receptors. The main trend of the receptor dynamics is specified by the positive feedback terms in the presence of both feedback loops, however, the negative feedback modulates the amplitude and position of the peaks and valleys. Consideration of positive and negative feedback loops and different time-delays adds extra parameters to signalling models via which the signalling can be regulated. Experimental data is required to estimate these parameters.

Parameters for TGF$-\beta$ Signalling Model

Tables 1, 2 and 3 show the parameters for all of the reactions for the RF- TGF$-\beta$ signalling model.

Supplementary Figures

Figure S3 shows the short-term (50 min) and long-term (500 min) responses of PSMAD concentration for different TGF$-\beta$ concentration. We have eliminated the negative feedback terms in the equations for R and RC in Figure S3, i.e. the negative feedback term appears only in the differential equation for PC. By comparing Figure S3 with Figure 5 in the numerical simulation section, we confirmed that the effects of the system's negative feedback is almost entirely on PC. The Hill coefficients of the transient and steady-state responses in Figure S3 are similar to the Hill coefficients of the corresponding curves in Figure 5.

Figure S4 shows the PSMAD responses in RF- model for different TGF$-\beta$ concentration at different times after TGF$-\beta$ stimulation (20, 50 200 and 500 minutes). The saturation level of PSMAD decreases as the simulation time increases. However, the Hill coefficient changes proportional to the simulation time. More specifically, the PSMAD response of the model does not switch before 200 minutes from the TGF$-\beta$ stimulation. This supports our hypothesis that the signal switching appears only in the long-term response of the cells to TGF$-\beta$ stimulation.

As a complementary Figure for Figure 8, Figure S5 shows PSMAD time-course of the RF- model for different SMAD concentrations. The production rate of SMAD (v_S) specifies the total SMAD concentration during the TGF$-\beta$ signalling. As v_S changes from 0.0005 to 0.05, the steady-state level of PSMAD increases until it reaches its saturation level (~ 0.75). Figure S5 shows that it takes longer for the

RF- model to reach its steady-state for higher SMAD production rates (v_{S}). Higher total SMAD concentration causes damped oscillations in the PSMAD time-course that last approximately for two days.

Figure S6 shows the experimental data used for validation of our model (see Figure 9). Similar to Figure 9, Figure S6.A represents the experimental time points from wild type MEFs and Figure S6.B shows the PSMAD2 protein level from Gp130$^{F/F}$ MEFs at different times after stimulation with TGF$-\beta$.

Figure S7 shows that the results of the RF- model of TGF$-\beta$ signalling are consistent with the experimental data from different cell lines (wild type MEFs and SV40-immortalized MEFs). Note that the solid curves are the PSMAD results predicted from the model. We have compared the output of the model with the experimental data for wild type and SV40 immortalised MEFs. Identical model parameters were used for the simulations drawn in late-stage tumor response in Figure 6, Figure S7.A and Figure S7.B (the solid curves). For the wild type MEFs (Figure S7.C), the model parameters were the same as Figure 4. It is clear that the model can follow the total PSMAD behaviour in time for both cell lines, however, the differences between the model simulation and the experimental data reveal that the RF- model should become re-parameterize when its simulations are compared to each set of experimental data (as is discussed and performed in section "Comparison of Simulation Results with Experimental Data").

Author details

References

1. Wagner, J., Keizer, J.: Effects of rapid buffers on Ca2+ diffusion and Ca2+ oscillations. Biophysical Journal **67**(1), 447 (1994)
2. Fall, C.P.: Computational Cell Biology (Interdisciplinary Applied Mathematics; V. 20), (2002). ISBN:0387953698
3. Ishikawa, H., Maeda, T., Hikita, H., Miyatake, K.: The computerized derivation of rate equations for enzyme reactions on the basis of the pseudo-steady-state assumption and the rapid-equilibrium assumption. Biochem. J **251**, 175–181 (1988)

Tables

Table 1: Components of the detailed TGF$-\beta$ signalling receptor model (Figure 3)

Components as variables	Components symbols in simulation and codes
TGF$-\beta$ receptor type 1	R1
TGF$-\beta$ receptor type 2	R2
TGF$-\beta$ receptor type 1 dimer	$(\mathrm{R1})_2$
TGF$-\beta$ receptor type 2 dimer	$(\mathrm{R2})_2$
Receptor complex	RC
Ligand-receptor complex	LC
Completely phosphorylated ligand-receptor complex	PC
Cytoplasmic SMAD	S
Phosphorylated SMAD in the cytoplasm	$\hat{\mathrm{S}}$
Phosphorylated SMAD in the nucleus	$\mathrm{S_n}$
Phosphorylated SMAD trimer	$(\mathrm{S})_3$
Delayed Phosphorylated SMAD trimer	N
Positive feedback intermediate inhibitor	P
Extracellular TGF$-\beta$ ligand	TGF$-\beta$ Dimer

Table 2: Kinetic rates and binding constants for the detailed TGF$-\beta$ signalling model (Figure 3)

Kinetic rates	Description	Unit
	TGF$-\beta$ Receptor	
v_1	Production rate of TGF$-\beta$ receptor type 1	$nMmin^{-1}$
k_1	Degradation rate of TGF$-\beta$ receptor type 1	min^{-1}
v_2	Production rate of TGF$-\beta$ receptor type 2	$nMmin^{-1}$
k_2	Degradation rate of TGF$-\beta$ receptor type 2	min^{-1}
k_1^+	Association rate of type 1 receptor homo-dimer complex	$nM^{-1}min^{-1}$
k_1^-	Dissociation rate of type 1 receptor homo-dimer complex	min^{-1}
k_2^+	Association rate of type 2 receptor homo-dimer complex	$nM^{-1}min^{-1}$
k_2^-	Dissociation rate of type 2 receptor homo-dimer complex	min^{-1}
k_{RC}^+	Association rate of receptor tetramer complex	$nM^{-1}min^{-1}$
k_{RC}^-	Dissociation rate of receptor tetramer complex	min^{-1}
k_{RC}	Degradation rate of receptor tetramer complex	min^{-1}
k_{LC}^+	Association rate of ligand-receptor complex	$nM^{-1}min^{-1}$
k_{LC}^-	Dissociation rate of ligand-receptor complex	min^{-1}
k_{PC}^+	Association rate of phosphorylated ligand-receptor complex	min^{-1}
k_{PC}^-	Dissociation rate of phosphorylated ligand-receptor complex	min^{-1}
k_{PC}	Degradation rate of phosphorylated ligand-receptor complex	min^{-1}
	SMAD Proteins	
v_{S}	Production rate of cytoplasmic SMAD	$nMmin^{-1}$
k_{S}	Degradation rate of SMAD in the cytoplasm	min^{-1}
k_{S}^+	Phosphorylation rate of SMAD in the cytoplasm	min^{-1}
k_{S}^-	Dephosphorylation rate of SMAD in the cytoplasm	min^{-1}
$k_{\mathrm{S_n}}$	Degradation rate of PSMAD in the nucleus	min^{-1}
k_n^+	Import rate of PSMAD into the nucleus	min^{-1}
k_n^-	Export rate of PSMAD from the nucleus	min^{-1}
k_3^+	Association rate of PSMAD homo-trimer complex	$nM^{-2}min^{-1}$
k_3^-	Dissociation rate of PSMAD homo-trimer complex	min^{-1}
K_{RC}	The receptor complex binding constant	nM
K_{S}	The phsphorylation binding constant	nM
K_3	PSMAD trimer binding constant	nM^2
	Feedback	
$k_1^{\mathrm{f}+}$	Positive feedback on TGF$-\beta$ receptor type 1	$nMmin^{-1}$
$k_1^{\mathrm{f}-}$	Negative feedback on TGF$-\beta$ receptor type 1	$nM^{-1}min^{-1}$
$k_{\mathrm{RC}}^{\mathrm{f}-}$	Negative feedback on receptor tetramer complex	$nM^{-1}min^{-1}$
$k_{\mathrm{PC}}^{\mathrm{f}-}$	Negative feedback on phosphorylated ligand-receptor complex	$nM^{-1}min^{-1}$
K	Negative feedback inhibitory binding constant	nM
K_{I}	Inhibition of the intermediate inhibitor binding constant	nM

Table 3: The parameter values of the RF- TGF$-\beta$ model (see Figure 3)

Parameters	Symbol	Literature value	Reference	Scaled data for our model
R production rate	v_1	0.0137 $nMmin^{-1}$	[68]	1 $nMmin^{-1}$
R degradation rate	k_1	0.00256 min^{-1}	[89, 19]	0.2 min^{-1}
R positive stimulated degradation rate	k_1^{f+}	$-$		1 min^{-1}
R negative stimulated degradation rate	k_1^{f-}	$-$		0.4 $nM^{-1}min^{-1}$
RC association rate	k_{RC}^{+}	$-$		0.5 $nM^{-1}min^{-1}$
RC dissociation rate	k_{RC}^{-}	$-$		0.5 min^{-1}
RC degradation rate	k_{RC}	$-$		1 min^{-1}
RC negative stimulated degradation rate	k_{RC}^{f-}	0.00256 $nM^{-1}min^{-1}$	[90]	0.4 $nM^{-1}min^{-1}$
PC association rate	k_{PC}^{+}	$-$		1.6 $nM^{-1}min^{-1}$
PC dissociation rate	k_{PC}^{-}	$-$		0.2 min^{-1}
PC degradation rate	k_{PC}	$-$		0.4 min^{-1}
PC negative stimulated degradation rate	k_{PC}^{f-}	$-$	-	0.4 $nM^{-1}min^{-1}$
S production rate	v_S	$-$		0.01 $nMmin^{-1}$
S degradation rate	k_S	$-$		0.008 min^{-1}
$\hat{S}$ association rate	k_S^{+}	0.049 $nM^{-1}min^{-1}$	[22]	0.1 min^{-1}
$\hat{S}$ dissociation rate	k_S^{-}	$-$		0.1 min^{-1}
$\hat{S}$ degradation rate	$k_{\hat{S}}$	0.394 min^{-1}	[20]	0.035 min^{-1}
S_n association rate	k_n^{+}	0.156 min^{-1}	[20, 19]	10 min^{-1}
S_n dissociation rate	k_n^{-}	0.739 min^{-1}	[20, 19]	8 min^{-1}
S_n degradation rate	$k_{\hat{S}}$	$-$		0.01 min^{-1}
positive/negative feedback constant	K	$-$		2
Inhibition of the intermediate inhibitor binding constant	K_I	$-$		0.4
SMAD phsphorylation binding constant	K_S	$-$		0.008
R initial value		$-$		0.83 nM
RC initial value		$-$		0.23 nM
S initial value		60.6 nM	[22]	1 nM
TGF$-\beta$ initial value		to be specified	[22]	to be specified
