# Supplementary figures and images for: Mathematical model of TGF-βsignalling: feedback coupling is consistent with signal switching
Source: BMC Syst Biol. 2017 Apr 13;11:48. doi: 10.1186/s12918-017-0421-5 (PMC5390422; doi:10.1186/s12918-017-0421-5)

Supplemental Figures S1-S7

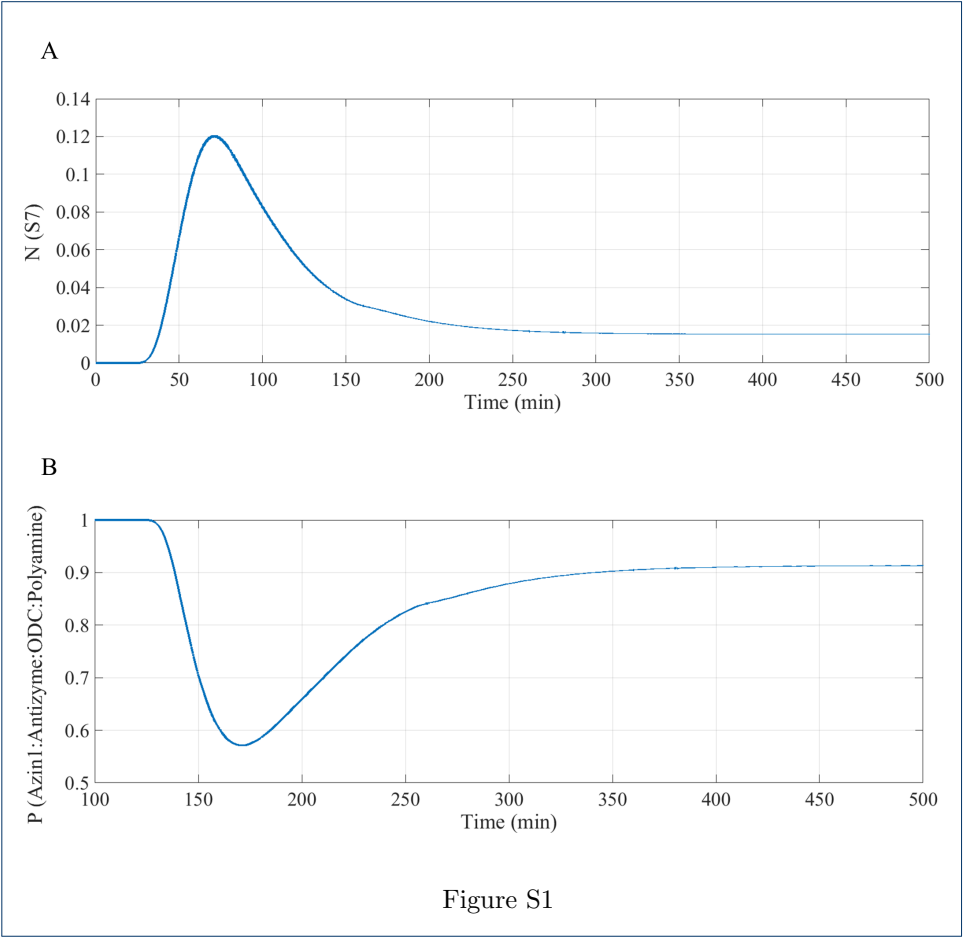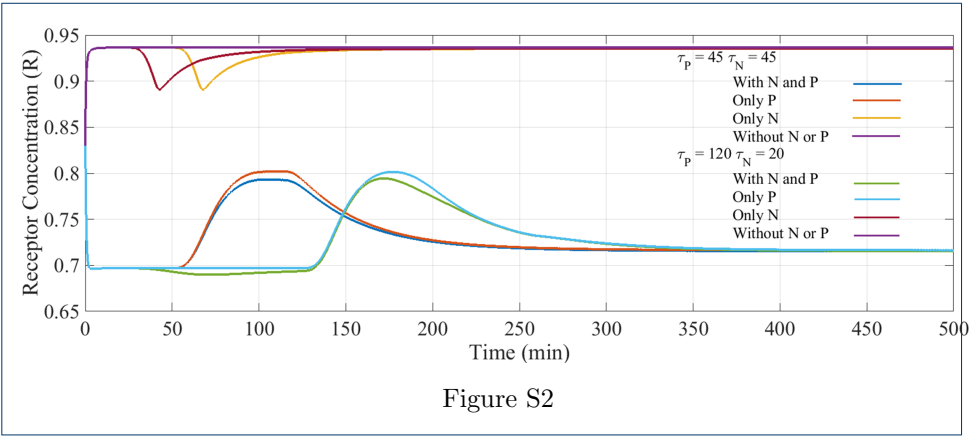

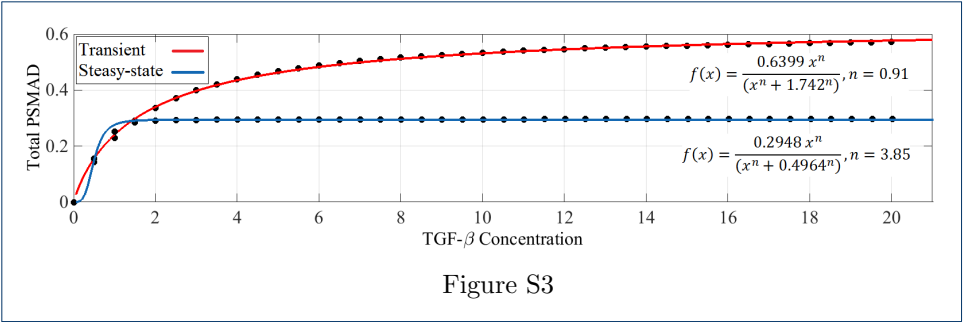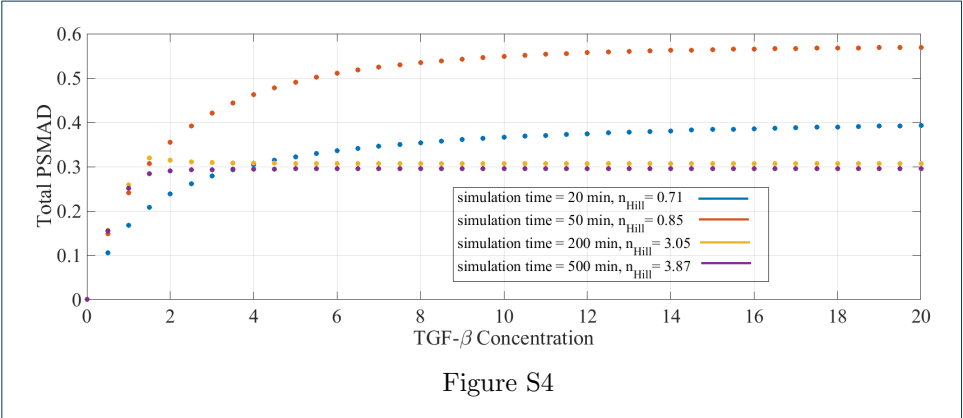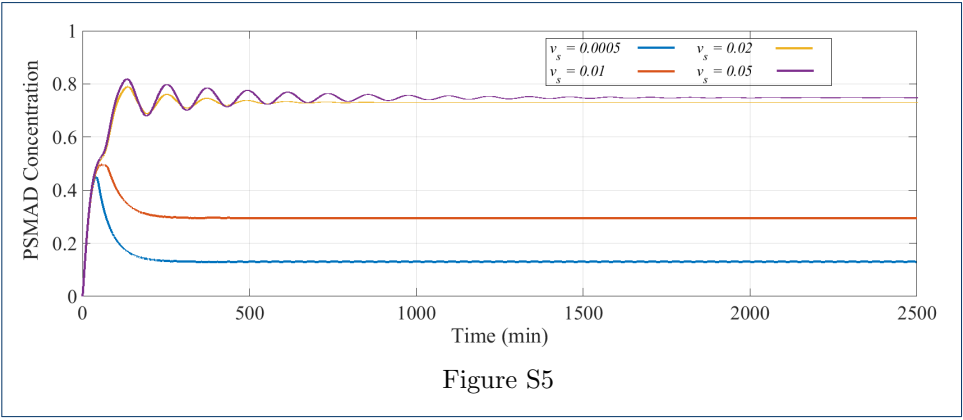

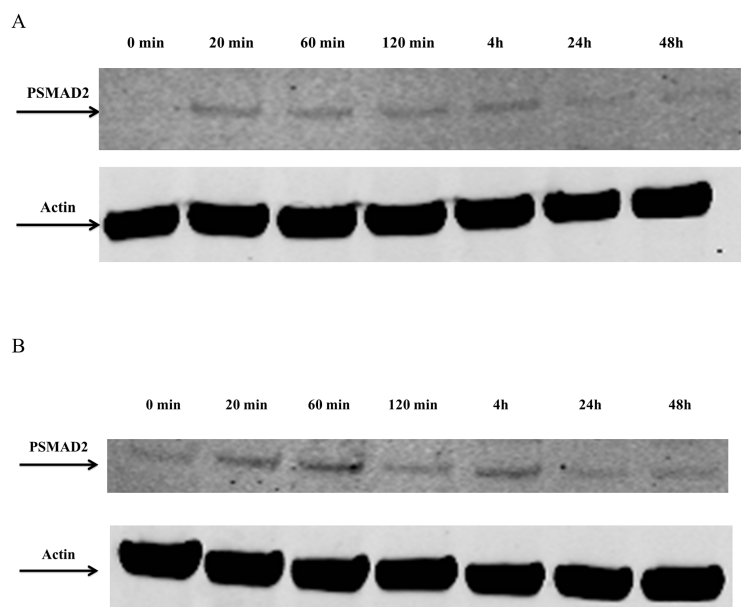

Figure S6

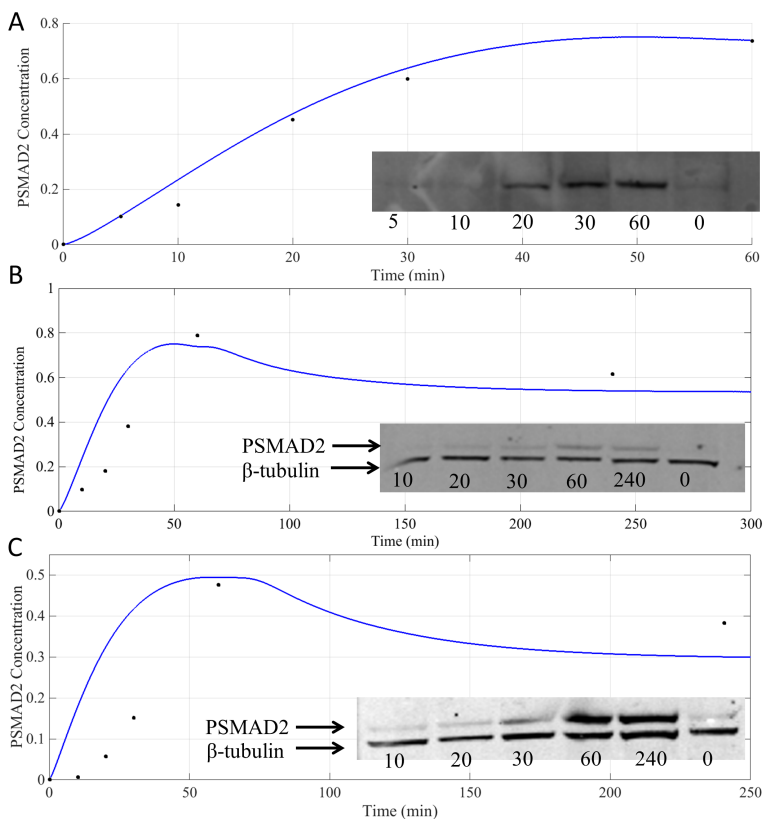

Figure S7

Supplement: Supplementary file 2 — Which provides information about extra figures (Figures S1-S7) of model simulation and experimental data that help understanding the signalling system. Figure S1 Dynamics of feedback loops in RF- model. A) The negative feedback loop time-course for a time-delay τ N=20 minutes B) The positive feedback loop time-course for a time-delay τ P=120 minutes. N changes proportionally with (S)3 as ((S)3) 2 while P is inversely proportional to (S)3 as 1/(1+((S)3)2). Figure S2 The effects of feedback loops on the TGF-βreceptor concentration dynamics. The time-delays are either for both τ N and τ P=45 minutes or τ P=120 and τ N=20 minutes. The effects of individual feedback loops on the receptor levels are studied. The peaks and the valleys are due to positive and negative feedback loops respectively. The time-delays shift the peaks and valleys in time. The strength of the feedback loops change the amplitude of the peaks and valleys. Figure S3 The predicted effects of different concentrations of TGF-β on PSMAD levels when the negative feedback loop influences PC only. These effects are shown for the short-term (50 min) and long-term (500 min) responses of the TGF-βsignalling system. Note that the dots are derived from model simulation and the curves show the Hill equations which are fitted by MATLAB. Figure S4 PSMAD responses to changes in TGF-β concentration at different simulation times. The Hill coefficient increases with the increase in the simulation time. The PSMAD response does not switch before 200 min, where the PSMAD level starts saturating (Fig. 4). The curves of 50 min and 500 min simulation times correspond to the curves in Fig. 5. Figure S5 PSMAD time-course for different production rates of SMAD The SMAD production rate (v S) determines the steady-state of the PSMAD response of RF- model. Higher v S SMAD concentration during the signalling. The PSMAD time-course experiences damped oscillation for high v S. The oscillations appear to delay the system reaching its s [file 12918_2017_421_MOESM2_ESM.pdf]
